# Identification of Meflin as a Potential Marker for Mesenchymal Stromal Cells

**DOI:** 10.1038/srep22288

**Published:** 2016-02-29

**Authors:** Keiko Maeda, Atsushi Enomoto, Akitoshi Hara, Naoya Asai, Takeshi Kobayashi, Asuka Horinouchi, Shoichi Maruyama, Yuichi Ishikawa, Takahiro Nishiyama, Hitoshi Kiyoi, Takuya Kato, Kenju Ando, Liang Weng, Shinji Mii, Masato Asai, Yasuyuki Mizutani, Osamu Watanabe, Yoshiki Hirooka, Hidemi Goto, Masahide Takahashi

**Affiliations:** 1 Department of Pathology, 65 Tsurumai-cho, Showa-ku, Nagoya 466-8550, Japan; 2 Department of Gastroenterology, 65 Tsurumai-cho, Showa-ku, Nagoya 466-8550, Japan; 3 Department of Physiology, 65 Tsurumai-cho, Showa-ku, Nagoya 466-8550, Japan; 4 Department of Nephrology, 65 Tsurumai-cho, Showa-ku, Nagoya 466-8550, Japan; 5 Department of Hematology and Oncology, Nagoya University Graduate School of Medicine, , 65 Tsurumai-cho, Showa-ku, Nagoya 466-8550, Japan; 6 Tumour Cell Biology Laboratory, The Francis-Crick Institute, 44 Lincoln’s Inn Fields, London, WC2A 3LY, United Kingdom

## Abstract

Bone marrow-derived mesenchymal stromal cells (BM-MSCs) in culture are derived from BM stromal cells or skeletal stem cells. Whereas MSCs have been exploited in clinical medicine, the identification of MSC-specific markers has been limited. Here, we report that a cell surface and secreted protein, Meflin, is expressed in cultured MSCs, fibroblasts and pericytes, but not other types of cells including epithelial, endothelial and smooth muscle cells. *In vivo*, Meflin is expressed by immature osteoblasts and chondroblasts. In addition, Meflin is found on stromal cells distributed throughout the BM, and on pericytes and perivascular cells in multiple organs. Meflin maintains the undifferentiated state of cultured MSCs and is downregulated upon their differentiation, consistent with the observation that Meflin-deficient mice exhibit increased number of osteoblasts and accelerated bone development. In the bone and BM, Meflin is more highly expressed in primitive stromal cells that express platelet-derived growth factor receptor α and Sca-1 than the Sca-1-negative adipo-osteogenic progenitors, which create a niche for hematopoiesis. Those results are consistent with a decrease in the number of clonogenic colony-forming unit-fibroblasts within the BM of Meflin-deficient mice. These preliminary data suggest that Meflin is a potential marker for cultured MSCs and their source cells *in vivo*.

Bone marrow-derived mesenchymal stromal cells (BM-MSCs), also termed mesenchymal stem cells, were originally identified as colony-forming unit-fibroblasts (CFU-Fs) in cultured BM cells^1^,^2^,^3^,^4^. Although the native identity and origin of BM-MSCs are not completely understood, recent evidence suggests that they are derived from bone marrow stromal cells (BMSCs) and skeletal stem cells (SSCs) that are located adjacent to BM sinusoids and arterioles and are essential for the development, postnatal remodeling and regeneration of bones^2^,^5^,^6^,^7^,^8^. The BMSCs/SSCs also constitute the niche for hematopoietic stem cells (HSCs), where they promote HSC maintenance by producing chemokine (C-X-C motif) ligand 12 (CXCL12) and stem cell factor (SCF, also known as c-kit ligand)^9^,^10^,^11^,^12^,^13^.

In culture, BM-MSCs exhibit multipotential differentiation capacity including osteogenic, chondrogenic and adipogenic lineages. They also possess trophic and immunomodulatory activities when they are transplanted or systemically infused into mammals^3^,^4^,^14^,^15^. Multilineage differentiation has also been observed in fibroblastic cells isolated from virtually every tissue, and they are referred to as MSCs, although the *in vivo* significance of the differentiation capacity has not been proven^16^. Cumulative evidence has shown that MSCs in culture originate from perivascular cells such as pericytes and perivascular fibroblasts^17^,^18^,^19^,^20^, which is reminiscent of the perisinusoidal location of BMSCs/SSCs in the BM. However, the extent to which perivascular cells are populated by MSCs *in vivo* is uncertain^19^. Also, the ontogenic relationship between BMSCs/SSCs in the BM and the perivascular cells in multiple organs has remained an issue^5^,^19^.

MSCs in culture are defined by the expression of cell surface markers such as CD73 (5′-ectonucleotidase), CD90 (Thy-1), CD105 (endoglin) and the absence of hematopoietic markers as well as HLA-DR, a major histocompatibility complex antigen^21^,^22^. Other markers have been also used for prospective isolation of distinct subpopulations of MSCs from various source tissues, including platelet-derived growth factor receptor α (PDGFRα), Sca-1, Stro-1, CD271 (low-affinity nerve growth factor receptor), CD106 (vascular cell adhesion molecule 1), CD146 (melanoma cell adhesion molecule), and others^21^,^23^. Studies on transgenic or knock-in mouse lines expressing reporter genes and lineage tracing approaches have revealed that BMSCs/SSCs can be defined by the leptin receptor (Lepr), CXCL12, gremlin 1, SCF, Mx1, and the nestin-GFP transgene^7^,^8^,^11^,^12^,^13^,^24^,^25^. Importantly, there is no known single molecular marker that unequivocally identifies MSCs and their descendants and distinguishes them from other cell lineages^11^,^21^. Moreover, the known markers of MSCs are not stable in their expression, as they depend on the developmental context and *in vitro* culturing^26^.

Through unrelated investigations, we came upon on a new cell surface protein that we termed “Meflin”, the function of which had not been addressed. Here we demonstrate that Meflin was expressed in cultured MSCs and was also detected sporadically *in situ* in the BM and perivascular regions in many types of organs. Our biochemical studies and results from Meflin-deficient mice showed that Meflin regulated the undifferentiated state of MSCs, suggesting that Meflin is useful for the detection of MSCs and their immature progeny both *in vitro* and *in vivo*.

## Results

### Meflin was expressed by the adipogenic cell line 3T3-L1 in superconfluent cultures and expression by cultured MSCs was dependent on population density

**Figure 1:**
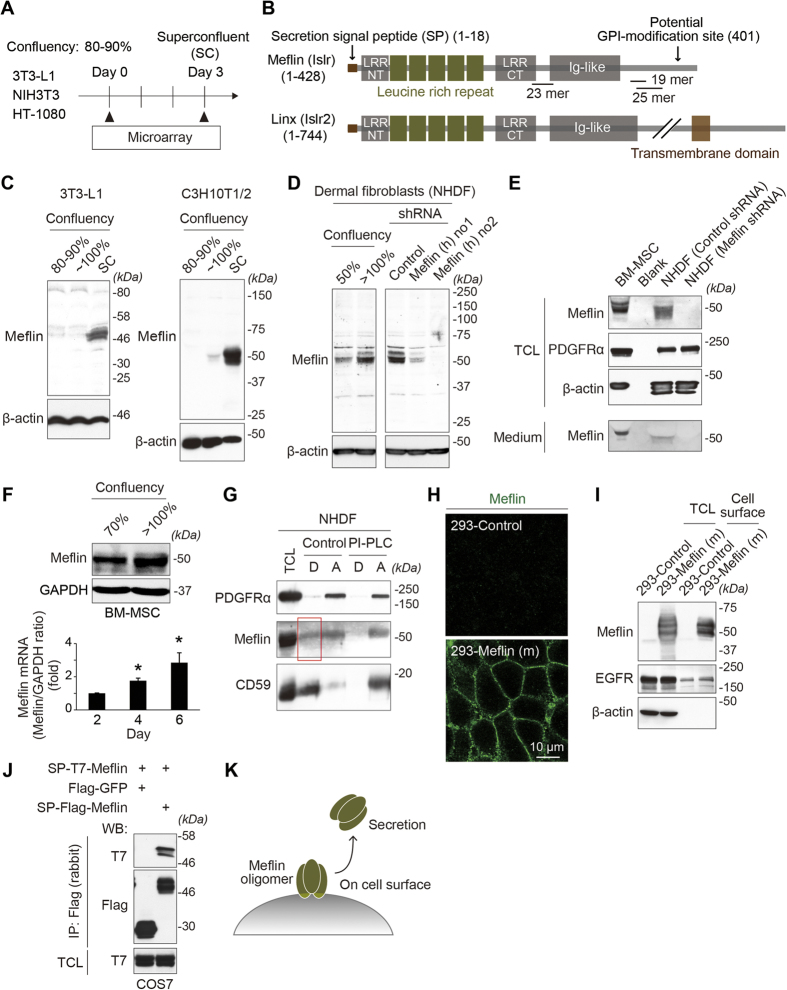
Meflin resided on the cell surface and was secreted by cultured fibroblasts and BM-MSCs. **(A)** Microarray analysis for the identification of upregulated genes in superconfluent (SC) 3T3-L1 and NIH3T3 fibroblasts, but not HT-1080 fibrosarcoma cells. **(B)** The primary domain structure of Meflin (Islr) and its paralogue Linx (Islr2). Locations of epitopes for the generation of Meflin antibodies (19 mer, 23 mer and 25 mer) are also shown. Numbers in parentheses indicate the number of amino acid residues. **(C)** Meflin was expressed specifically in contact-inhibited and superconfluent 3T3-L1 and C3H10T1/2 cells. kDa, kilodaltons. **(D–F)** Meflin protein was expressed in cultured dermal fibroblasts and BM-MSCs, depending on cell confluency. For the depletion of Meflin, cells were infected with retrovirus encoding the indicated shRNA followed by selection for puromycin. Note that Meflin was secreted into the medium **(E)**. In the lower panel of **(F)**, Meflin mRNA was measured by qPCR every two days after plating 2 × 10^5^ BM-MSCs in 3.5-cm dishes. TCL, total cell lysates. **(G)** Meflin identified as a GPI-anchored protein. Proteins extracted from fibroblasts by Triton X-114 in the presence or absence of phosphatidylinositol-specific phospholipase C (PI-PLC) were tested by Western blot analysis, where CD59 was used as a positive control. The red box indicates GPI-anchored Meflin that is sensitive to PI-PLC treatment. D, detergent phase; A, aqueous phase. **(H)** 293 cells that stably expressed mouse (m) Meflin (lower panel) and control cells (upper panel) were stained with the anti-Meflin antibody. **(I)** Isolation of cell surface proteins by biotin labeling from control and 293-Meflin cells, showing that Meflin was predominantly expressed on the cell surface. **(J)** T7-tagged Meflin was cotransfected with Flag-tagged Meflin into COS7 cells, followed by immunoprecipitation by anti-Flag antibody and Western blot analysis with indicated antibodies, showing that Meflin formed an oligomer. **(K)** Meflin, a cell surface oligomeric protein, was also secreted by unknown mechanisms.

Our initial aim was to investigate the mechanism of contact inhibition of proliferation and locomotion, a characteristic of normal cells that is lost in malignant cells. We used microarray analysis of representative non-transformed fibroblasts (3T3-L1 and NIH3T3) and compared gene expression profiles between subconfluent (80–90%) and superconfluent (>100%) monolayer cultures of those lines and a malignant fibrosarcoma cell line HT-1080 (Fig. 1A). Among the upregulated (Table S1) and downregulated (data not shown) genes (changed at least 4-fold) in both contact-inhibited 3T3-L1 and NIH3T3 but not HT-1080 cells, we focused on a gene that was 7.84-, 5.09- and 0.38-fold changed in 3T3-L1, NIH3T3 and HT-1080, respectively. The gene encoded an immunoglobulin superfamily-containing leucine-rich repeat (*Islr*), the function of which was not known^27^. Islr is a member of the leucine-rich repeat and immunoglobulin (LIG) family of proteins^28^ and a paralogue of Linx (also termed Islr2) that has important roles in the development of the central and peripheral nervous system (Fig. 1B)^29^,^30^.

Our subsequent experiments showed that Islr protein did not have any roles in either contact inhibition of proliferation or locomotion (data not shown), leading to the speculation that Islr was linked to other cellular processes. A previous study that comprehensively investigated the expression of the members of the LIG family by *in situ* hybridization (ISH), showed that *Islr* was exclusively expressed in the mesenchyme in the head, trunk, and limbs in developing mouse embryos, which is in stark contrast to Linx/Islr2 that was specifically expressed in neural tissues^31^. Also, a survey of gene expression studies provided evidence that *Islr* expression was at high levels in cultured BM-MSCs and adipose tissue-derived stem cells (ADSCs)^32^,^33^,^34^,^35^, but not in neural or embryonic stem cells^36^. On the basis of these and subsequent findings, we renamed the protein encoded by the *Islr* gene “Meflin (mesenchymal stromal cell- and fibroblast-expressing Linx paralogue)”. Meflin is comprised of a secretion signal peptide (SP) at the amino (N)-terminal end, five tandemly linked leucine-rich repeat (LRR) domains flanked by LRR N- and carboxyl (C)-terminal cysteine-rich domains, and an immunoglobulin-like domain (Figs 1B, S1). Consistent with the microarray analysis, Western blot analysis using antibodies generated in this laboratory showed that Meflin was expressed in superconfluent and contact-inhibited 3T3-L1 (Fig. 1C). Meflin was also detected in superconfluent C3H10T1/2, a cell line with characteristics of MSCs (Fig. 1C). In contrast, Meflin was constitutively expressed in primary dermal fibroblasts, BM-MSCs, and ADSCs, the extent of which largely depended on the extent of cell confluency, implying a link between cell cycle regulation and Meflin expression (Figs 1D–F, S2). In these experiments, the specificity of the Meflin antibodies was shown by short hairpin RNA (shRNA)-mediated depletion of Meflin (Fig. 1D,E). In a survey of different cell types, Meflin was not detected in epithelial, endothelial, smooth muscle, or cancer cells (Fig. S2).

Consistent with the presence of a potential glycosyl-phosphatidylinositol (GPI)-modification site at the C-terminal end of Meflin (Figs 1B, S1), our biochemical analysis showed GPI-modification of at least some populations of Meflin (Fig. 1G), which was further supported by immunostaining and biochemical analysis showing its localization on the cell surface (Fig. 1H,I). Similar to other members of the LIG family of proteins, Meflin has the capacity to form an oligomer, although the significance of the oligomerization is unclear at present (Fig. 1J). Meflin was also detected in spent culture media from BM-MSCs and fibroblasts (Figs 1E, S2), indicating that Meflin undergoes some cleavage processes or secretion machinery (Fig. 1K).

### Meflin was expressed in the skeletal tissues of embryos and in the BM and adipose tissues of adult mice

**Figure 2:**
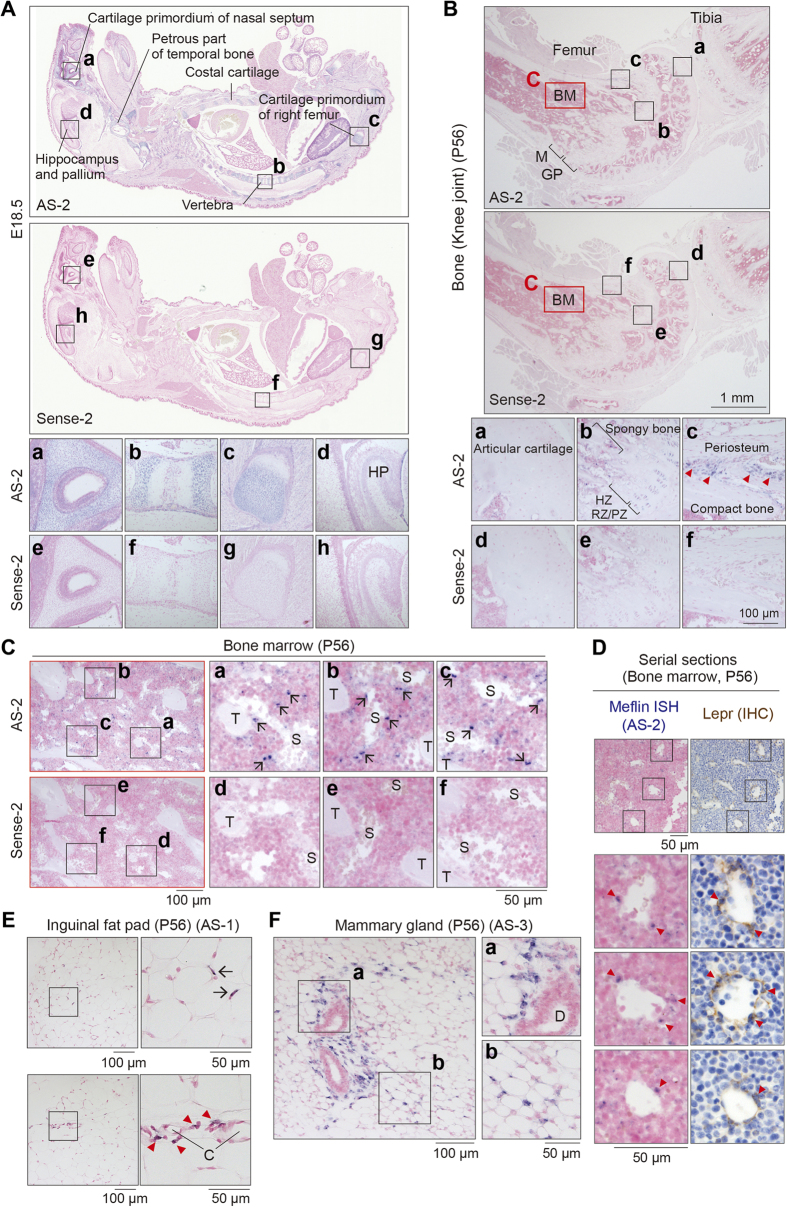
Expression pattern of Meflin in mouse tissues. **(A)** ISH analysis with Meflin antisense (AS) and control (Sense) probes. Box regions are magnified in adjacent panels (a–h). The data show the expression of Meflin in the cartilage primordia of nasal septum (a,e), temporal bone, costal cartilage, vertebra (b,f), and femur (c,g) in E18.5 embryos. Neural tissues such as pallium are almost negative for Meflin, with an exception that the hippocampus (HP) shows marginal expression of Meflin (d,h). **(B)** Meflin expression in the knee joint in adult (P56) mice. Meflin was expressed in the resting and proliferative zone (RZ/PZ), but not hypertrophic zone (HZ) of the growth plate (GP) (b,e). Meflin was also expressed in cells condensing near the periosteum (red arrowheads) (c,f). No Meflin expression was detected in mature chondrocytes in the articular cartilage (a,d) or in osteocytes in the compact bone (c,f). Red box regions (BM) are magnified and described in **(C)**. M, metaphysis. **(C)** The magnification of the BM region in **(B)** shows Meflin expression in cells that are sporadically distributed in the BM. The majority of Meflin^+^ cells (arrows) was detected in the perisinusoidal region, whereas some were in the peritrabecular region. T, trabeculae; S, sinusoids. **(D)** ISH for Meflin (left) and immunohistochemistry (IHC) for Lepr (right) on serial sections from the BM showed partial coexpression of Meflin and Lepr in stromal cells around the sinusoids (red arrowheads). **(E**,**F)** Meflin expression in adipose tissues. Meflin^+^ cells were sparsely detected in the adipose tissues of inguinal **(D)** and mammary **(E)** fat pad regions (arrows). Note that some of the Meflin^+^ cells were detected in perivascular regions (red arrowheads) and periductal regions in the inguinal fat pad and the mammary fat pad, respectively. C, capillaries; D, milk duct.

Our ISH study (Fig. 2A) revealed the expression of Meflin in cells that constitute the stroma and the cartilage primordia of skeletal tissues in mouse embryos, consistent with findings in the previous study^31^. In the bones of adult mice at postnatal (P) day 56, Meflin was detected in immature chondroblasts in the resting and proliferative zones of the growth plate (GP), but not mature chondrocytes in the hypertrophic zone or the articular cartilage (Fig. 2B). Meflin was also expressed in cells condensing around the periosteum; they appeared to be immature osteoblasts and not mature osteocytes that constitute compact bone (Fig. 2B).

An intriguing finding was that Meflin-positive (Meflin^+^) cells were sporadically distributed and scattered throughout the BM, and many of them were located adjacent to the perisinusoidal area, whereas others were near the peritrabecular area (Fig. 2C). The frequency of Meflin^+^ cells throughout the BM was estimated to be less than a few percent of all nucleated cells, leading to the notion that Meflin defines a rare population of BM cells and not an abundant hematopoietic lineage. Evidence supporting this was obtained from the expression database of mouse HSCs and their differentiated progeny^37^ (Gene Expression Omnibus accession number GSE6506), which showed that Meflin expression was not detected in either HSCs or any hematopoietic lineage (Fig. S3). Moreover, another study^13^ and its accompanying microarray analysis (GSE33158) showed that Meflin expression was highly enriched in SCF-positive BMSCs, which is nearly equivalent to Lepr-expressing cells^8^ or CXCL12-abundant reticular (CAR) cells^12^, but it was not detected in whole BM cells (Fig. S4). Consistent with this, our ISH and immunohistochemical analysis of serial sections prepared from the BM showed that Meflin^+^ cells partially overlapped with the population of cells expressing Lepr around the sinusoids (Fig. 2D). These data implied that Meflin was expressed in BMSCs/SSCs but not hematopoietic lineage cells in the BM, where it may be involved in the formation of the hematopoietic microenvironment.

Other sites where Meflin^+^ cells were detected included adipose tissue in the mammary gland and the inguinal fat pad. In the adipose tissues, Meflin^+^ cells were sparse among mature adipocytes and the perivascular region (Fig. 2E). In the mammary gland, some cells around the milk ducts, which appeared to be fibroblasts, were positive for Meflin (Fig. 2F). Throughout the ISH study, in which we confirmed data reliability by using three independent probes (see Supplementary Information), Meflin expression was not detected in epithelial, endothelial or neural cells. One exception was the developing mouse embryo in which the hippocampus was weakly positive for Meflin, although we are at present unaware of the significance of such minimal staining (Fig. 2Ad). These expression data are consistent with the selective expression of Meflin in cultured BM-MSCs and ADSCs but not other types of cells (Figs 1E,F, S2), implying an *in vivo* role of Meflin in MSCs and their early descendants.

### Expression of Meflin in perivascular and stromal cells in various tissues

**Figure 3:**
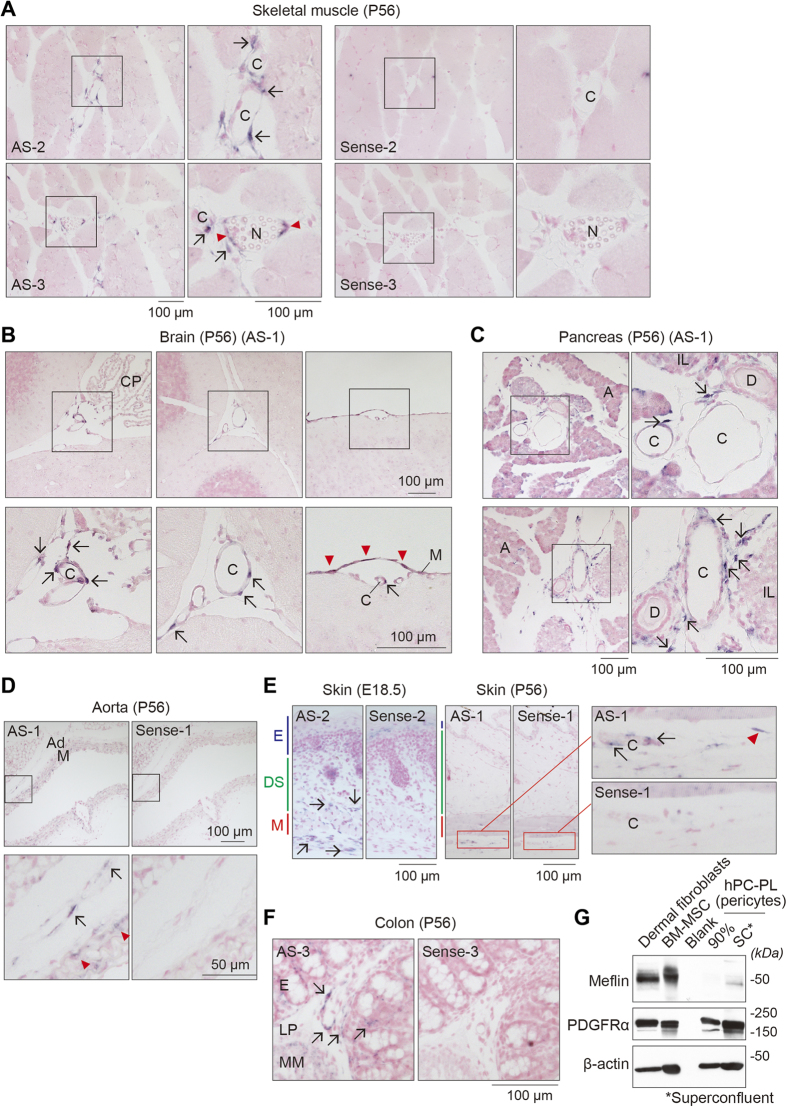
Meflin expression in perivascular, perineurium, and meningothelial cells, and reticular fibroblasts. **(A)** Meflin expression in subendothelial pericytes and perivascular fibroblasts (arrows) around the capillaries (C) and perineurium cells (arrowheads) around the nerve (N) among the muscular bundles. **(B)** Meflin expression in pericytes in the subarachnoid cavity (arrows) and meningothelial cells (arrowheads) in the meninges (M) in the adult brain. Note that Meflin expression was neither found in the epithelial cells in the choroid plexus (CP) nor neurons in the brain parenchyma. **(C)** In the pancreas, Meflin was expressed in perivascular and periductal fibroblasts, but not in the constituents of the islands of Langerhans (IL) nor the acini (A). D, interlobular duct; C, capillaries. **(D)** In the abdominal aorta, Meflin was expressed in fibroblasts (arrows) in the adventitia (Ad). Note that Meflin was not expressed in smooth muscle cells in the tunica media (M), despite the weak expression of Meflin in some cells found in the outer layer of the tunica media (arrowheads). **(E)** Meflin expression in the skin. In the skin of the back of mouse embryos (left panels), Meflin was expressed in fibroblasts (arrows) in and around the subcutaneous muscle layer (M) but was very rare in the dermis and subcutaneous tissues (DS) and the epidermis (E). In adult skin (right panels), Meflin expression was detected in pericytes (arrows in the magnified region) and fibroblasts (arrowhead) around the subcutaneous muscle. **(F)** Meflin expression in the colon. Meflin was detected in reticular fibroblasts (arrows) in the lamina propria (LP) in the mucosa. E, epithelium; MM, muscularis mucosa. **(G)** Western blot analysis shows Meflin expression in superconfluent (SC), but not 90% confluent, primary pericytes isolated from the placenta. Dermal fibroblasts and BM-MSCs serve as positive controls.

In adipose tissue, we located Meflin^+^ cells in perivascular areas around microvessels and capillaries, some of which seemed to localize in the periendothelial compartments or make close contact with the abluminal membrane of endothelial cells (Fig. 2E). Of note, not all of the perivascular cells were positive for Meflin, reflecting the heterogeneity of those cells^20^. The expression of Meflin in the perivascular cells was also observed across various tested organs, including skeletal muscle, brain, pancreas and skin (Fig. 3A–F), data that were consistent with previous studies showing that MSCs in culture originate from or share properties with some pericytes or perivascular fibroblasts^18^,^19^. Our Western blot analysis showed a modest expression of Meflin in cultured pericytes when they were superconfluent, indicating that some (but not all) populations of pericytes expressed Meflin (Fig. 3G). Taken together, the data led to the speculation that Meflin essentially marked two populations of cells: (1) BMSCs/SSCs-lineage immature cells in the bone and (2) perivascular and stromal cells in other organs.

Other Meflin^+^ cells included perineurium cells around nerves in skeletal muscle (Fig. 3A), meningothelial cells in the brain (Fig. 3B), stromal cells around the epithelial ducts in the pancreas (Fig. 3C) and fibroblasts in the adventitia of the aorta (Fig. 3D). Smooth muscle cells that surrounded large-sized vessels were negative for Meflin, with one exception that the branches of renal arteries comprised Meflin^+^ cells in a mosaic-like manner (data not shown). Meflin was also expressed by the reticular fibroblasts in the interstitium of the lamina propria in the colon, the localization pattern of which is reminiscent of reticular stem cells in the intestine (intestinal reticular stem cells; iRSCs) that were recently identified by the expression of gremlin 1 (Fig. 3F)^24^.

In skeletal muscle, Meflin expression was also detected in single cells located at the edges of muscle fibers, which is reminiscent of satellite cells or myogenic precursors that contribute to the growth and regeneration of the muscle^38^, although the identity of the Meflin^+^ cells remains uncertain (Fig. S5). We enzymatically fractionated non-hematopoietic (CD45^−^Ter119^−^) mononuclear cells from hind limb muscle. We found that Meflin was expressed in PDGFRα^+^ cells that represent mesenchymal progenitors located in the muscle interstitium and play an important role in muscle homeostasis^39^. Meflin was expressed at a lesser extent in PDGFRα^−^ cells that comprise satellite cells or other myogenic precursors (Fig. S5). Together with the ISH study, the data suggested that Meflin was expressed in different types of cells in skeletal muscle.

### Meflin defined the undifferentiated state of cultured BM-MSCs and C3H10T1/2 cells

**Figure 4:**
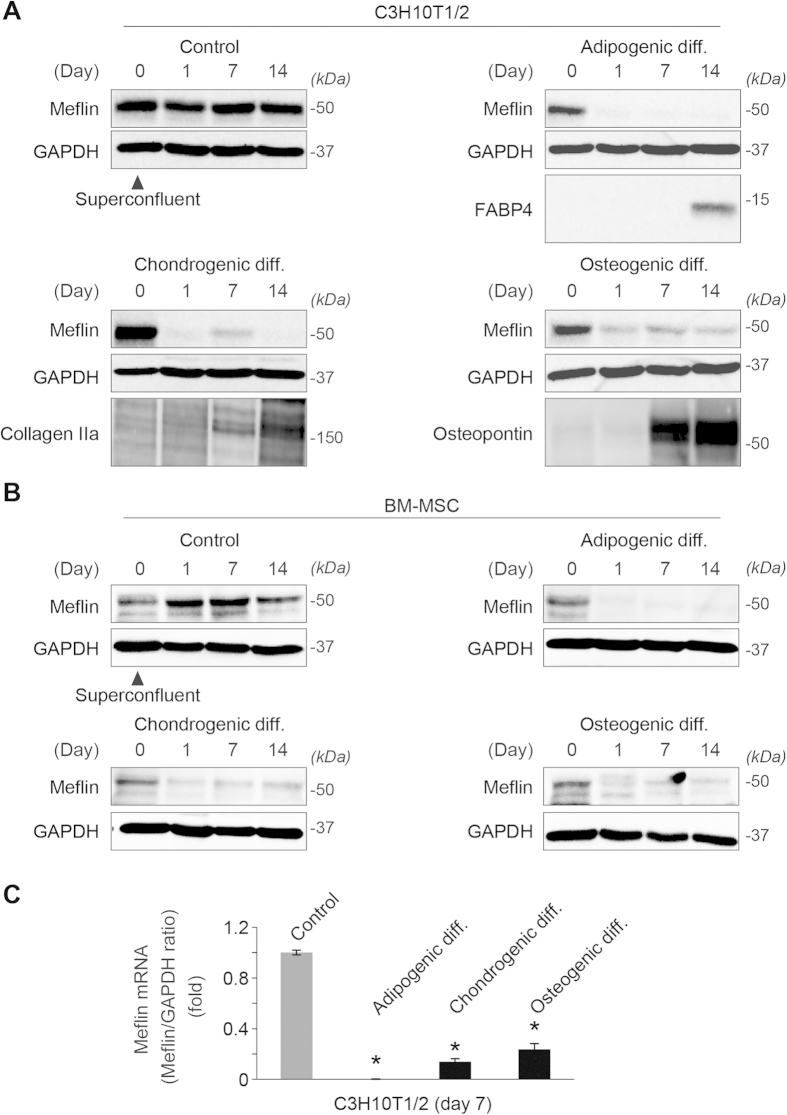
Downregulation of Meflin in the differentiation of MSCs. **(A**,**B)** Western blot analysis showed the downregulation of Meflin one day after the initiation of adipogenic, chondrogenic, and osteogenic differentiation of C3H10T1/2 **(A)** and BM-MSCs **(B)**. FABP4, fatty acid binding protein-4. **(C)** qPCR showed the downregulation of mRNA for Meflin in the trilineage differentiation of C3H10T1/2 cells. *P < 0.05 compared with control.

**Figure 5:**
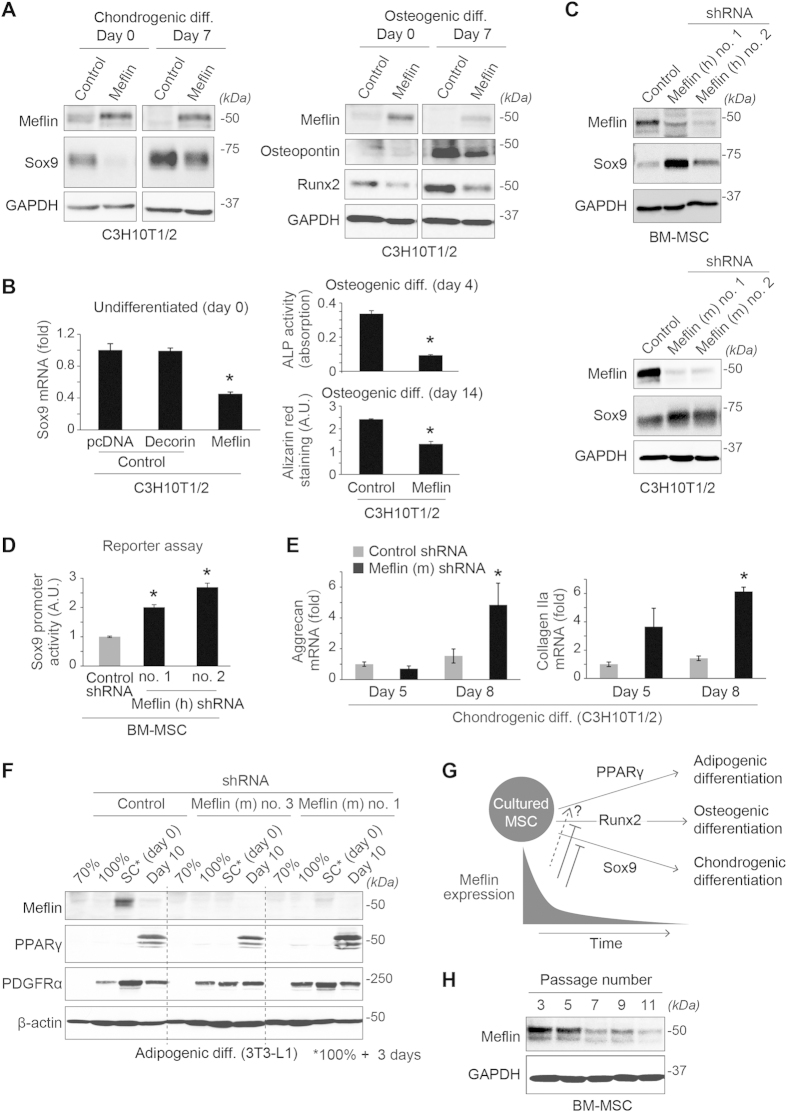
Meflin regulated undifferentiated state of C3H10T1/2 cells and BM-MSCs. **(A)** Forced exogenous expression of Meflin suppressed the expression of Sox9 protein in chondrogenic differentiation (left panel) and Runx2 and osteopontin proteins in osteogenic differentiation (right panel) in C3H10T1/2 cells. **(B)** Forced expression of Meflin, but not Decorin (control), led to the downregulation of basal expression of the *Sox9* gene in undifferentiated C3H10T1/2 cells (left panel). In cells that underwent osteogenic differentiation (right panel), Meflin suppressed alkaline phosphatase (ALP) activity and calcium deposit as determined by Alizarin red staining. **(C)** Meflin-depletion led to the upregulation of the basal expression of Sox9 protein in BM-MSCs (top panel) and C3H10T1/2 cells (lower panel). **(D)** Meflin-depletion upregulated the activity of the human *Sox9* promoter in BM-MSCs, as determined by luciferase reporter assay. *P < 0.05 compared with control. A.U., arbitrary units. **(E)** qPCR assay showed that Meflin-depletion led to the upregulation of Aggrecan and Collagen IIa, the hallmarks of chondrogenic differentiation, in C3H10T1/2 cells. *P < 0.05 compared with control. **(F)** No apparent effect of Meflin-depletion on adipogenic differentiation in 3T3-L1 cells. Note that Meflin expression was specifically detected in superconfluent (SC) cells that underwent rapid downregulation by adipogenic differentiation. **(G)** Schematic illustration of our preliminary hypothesis on the role of Meflin in determining the undifferentiated state of cultured MSCs. Meflin is expressed in undifferentiated MSCs to suppress the induction of Sox9 and Runx2 expression. At present, the role of Meflin in adipogenic differentiation remains undetermined. **(H)** Gradual decrease of Meflin expression depending on the passage number in BM-MSCs.

Monitoring the expression of Meflin during trilineage differentiation (osteogenic, chondrogenic, and adipogenic) of BM-MSCs and C3H10T1/2 cells, we found that its expression underwent immediate downregulation at both protein (Fig. 4A,B) and mRNA (Fig. 4C) levels on the first day after initiating differentiation. The data support the hypothesis that Meflin is involved in or maintains the undifferentiated state of MSCs in culture. This idea was supported by the finding that the exogenous expression of Meflin suppressed the expression of the Sox9 (SRY-related high-mobility group box 9) and Runx2 (runt-related transcription factor 2) proteins, master regulators for chondrogenic and osteogenic differentiation, respectively^40^, in the differentiation of C3H10T1/2 cells (Fig. 5A). Meflin also significantly suppressed the basal level of Sox9 mRNA in undifferentiated cells, as well as alkaline phosphatase (ALP) activity and calcium deposition during osteogenic differentiation (Fig. 5B). In addition, the depletion of endogenous Meflin led to the upregulation of Sox9 protein expression (Fig. 5C) and its promoter activity (Fig. 5D), as well as the expression of Aggrecan and Collagen IIa gene expression, hallmarks for chondrogenic differentiation (Fig. 5E). These data all suggest that Meflin was involved in the maintenance of the undifferentiated state of MSCs (Fig. 5G). Meflin-depletion had no apparent effect on the expression of peroxisome proliferator-activated receptor γ (PPARγ) protein, a master regulator of adipogenesis^41^, in 3T3-L1 cells, leaving the significance of Meflin in adipogenesis undetermined at present (Fig. 5F,G). We also found that Meflin protein expression was downregulated by continuous passage in culture (Fig. 5H) and by culture on stiff substrates that induce cellular traction forces and osteogenic differentiation (Fig. S6)^42^, further supporting the view that Meflin was involved in the maintenance of the undifferentiated state of MSCs.

### The function of Meflin was distinct from those of other members of the LIG family of proteins

**Figure 6:**
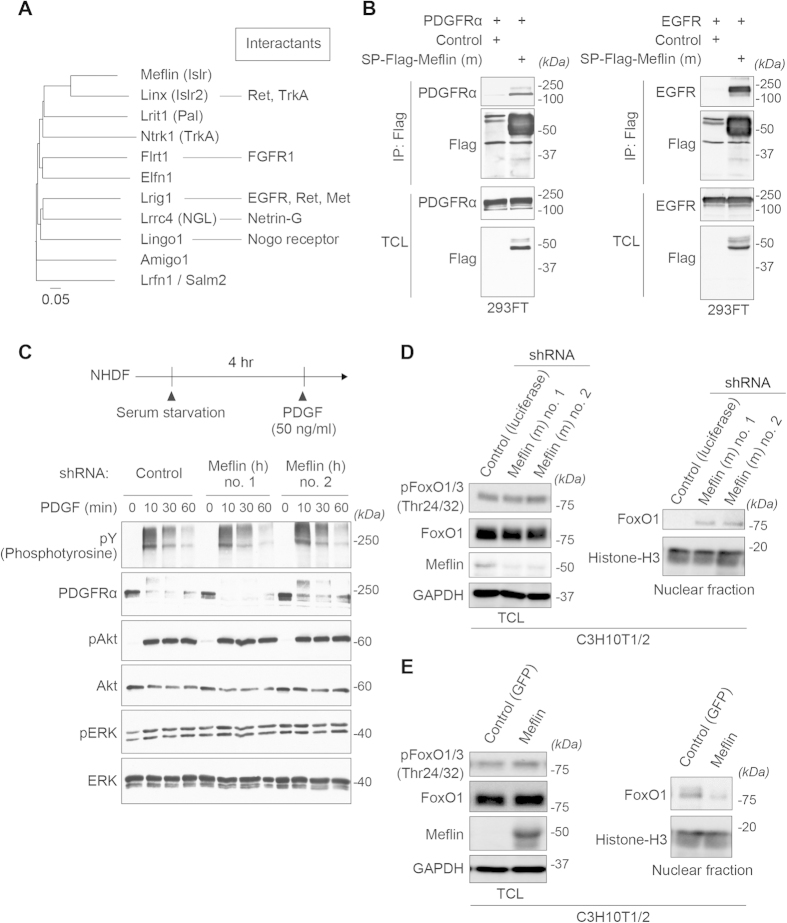
Meflin function was distinct from other members of the LIG family or proteins. **(A)** A phylogenetic tree showing the evolution of representative members of the LIG family of proteins. The scale bar indicates the rate of amino acid substitutions per site. The interacting proteins for each member of the LIG family, many of which are RTKs, are also shown. **(B)** Interaction of Meflin with PDGFRα (left panel) and EGFR (right panel). 293FT cells were transfected with the indicated plasmids, followed by immunoprecipitation (IP) and Western blot analysis. **(C)** No apparent effect of Meflin-depletion in PDGF signaling in dermal fibroblasts. Lysates from control and Meflin-depleted cells stimulated with recombinant rat PDGF-BB for indicated times were subjected to Western blot analysis using the indicated antibodies. **(D)** Meflin regulated nuclear localization of the FoxO1 transcription factor. Western blots were used to examine whole lysates (left panel) and nuclear fractions (right panel) isolated from C3H10T1/2 cells transduced with retroviruses expressing luciferase and Meflin shRNAs. **(E)** C3H10T1/2 cells were transduced with retroviruses expressing GFP (control) and Meflin, followed by Western blot analysis. Overexpression of Meflin suppressed the nuclear localization of FoxO1, without apparently affecting its phosphorylation. Histone-H3 is a marker for nuclear proteins.

A number of previous studies have shown that the other members of the LIG family of proteins, such as Linx/Islr2, leucine-rich repeats and immunoglobulin-like domains-1 (Lrig1), adhesion molecule with immunoglobulin like domain 1 (Amigo1), and fibronectin leucine rich transmembrane 1 (Flrt1), interact with receptor tyrosine kinases (RTKs) to negatively or positively regulate their downstream signaling for neural development, differentiation control of tissue stem cells and cancer progression^29^,^43^,^44^,^45^,^46^ (Fig. 6A). Indeed, our immunoprecipitation study showed the interaction of Meflin with RTKs such as epidermal growth factor receptor (EGFR) and PDGFRα (Fig. 6B). However, we found no apparent effect of Meflin-depletion on the downstream signaling from the RTKs when the activation of extracellular-signal-regulated protein kinase (ERK) and Akt was used as the readout (Fig. 6C). We also tested the possibility that secreted Meflin acted in trans on cell surface RTKs. Adding recombinant purified Meflin to fibroblasts, however, did not exert any effect on PDGF-mediated ERK/Akt activation (Fig. S7A,B). Contrary to previous studies that some LIG family members regulated cell proliferation^43^, Meflin-depletion had no apparent effect on cell proliferation (Fig. S7C,D). These data suggested that the function of Meflin may be different from other members of the LIG family of proteins.

A previous genome-wide screening study using the human U2OS osteosarcoma cell line identified Meflin *(Islr)* as one of the genes that regulated the nuclear localization of the forkhead box O1 (FoxO1) transcription factor^47^. Our fractionation study also demonstrated that Meflin-depletion led to the nuclear translocation of FoxO1 (Fig. 6D). Consistent with that was the inhibition of nuclear localization of FoxO1 by the overexpression of Meflin (Fig. 6E). FoxO1 regulates osteoblast differentiation and bone formation through interaction with the activating transcription factor 4 (ATF4) transcription factor and the promoter of the *Runx2* gene^48^,^49^. Thus, the data are in an agreement with the idea that Meflin is involved in the undifferentiated state of MSCs. Although the mechanism by which Meflin regulates the subcellular localization of FoxO1 remains unclear at present, the data revealed a novel feature of the LIG family of proteins that is distinct from the regulation of RTK signaling pathway.

### Meflin-deficiency led to the accelerated development of long bones and low CFU-F potential of BM cells

**Figure 7:**
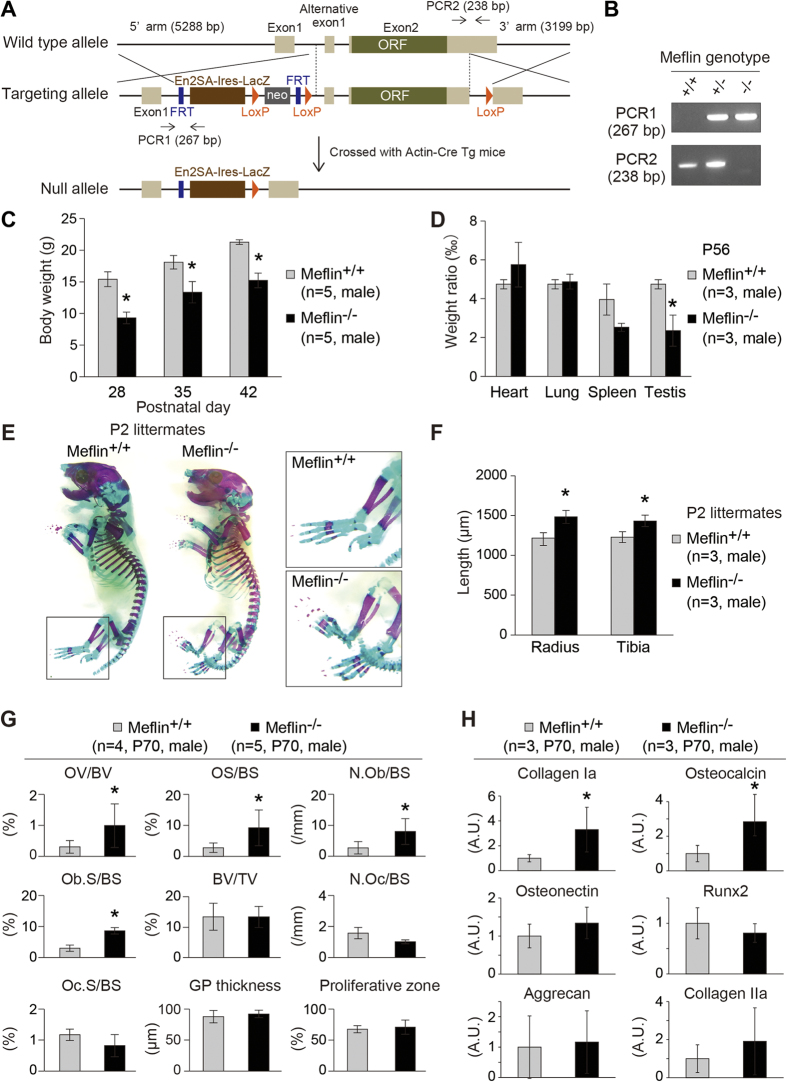
Meflin-deficiency led to aberrant development of bones. **(A)** A schematic illustration showing the strategy for targeting the Meflin (*islr*) gene, which was designed by the EuMMCR. Note that exon 2 of the Meflin (*islr*) gene encodes a whole open reading frame (ORF). The sites for PCR primers for genotyping are also shown. **(B)** A representative data of genotypic PCR shows the complete deletion of the WT alleles in Meflin^−/−^ mice. **(C**,**D)** The weights of whole bodies and the indicated organs harvested from P56 male mice were measured, indicating growth retardation in Meflin^−/−^ mice. In **(D)**, the weight of each organ was normalized by body weight (n = 3). *P < 0.05 compared with WT mice. **(E)** Representative images of the skeletons of a Meflin^−/−^ P2 male mouse and its WT littermate are shown. Boxed areas are magnified in adjacent panels. **(F)** Quantification of the lengths of the radii and tibiae from WT and Meflin^−/−^ P2 littermates (n = 3). *P < 0.05 compared with WT mice. **(G)** Bone histomorphometric analyses of the secondary spongiosa area and the GP of tibiae from WT and Meflin^−/−^ P70 littermates (n = 4 and 5, respectively). Note a significant increase in osteoblast number/bone surface (N.Ob/BS), osteoblast surface/bone surface (Ob.S/BS), osteoid surface/bone surface (OS/BS) and osteoid volume/bone volume (OV/BV) in Meflin^−/−^ mice. *P < 0.05 compared with WT mice. No apparent differences were seen in the thickness of the GP and the proliferative zone between WT and Meflin^−/−^ P70 mice. **(H)** Quantitation of the expression of osteogenic and chondrogenic genes in the tibiae from WT and Meflin^−/−^ P70 mice by qPCR (n = 3). *P < 0.05 compared with WT mice. The data are presented as the fold-increase compared with WT mice.

In view of the results presented above, we generated and analyzed Meflin-deficient (^−/−^) mice (Fig. 7A,B). Meflin^−/−^ mice were born at a ratio predicted by Mendelian genetics without any gross abnormal findings, whereas they gradually showed growth retardation after their birth as measured by whole body weights and the testis (Fig. 7C,D). Despite the observed growth retardation, the analysis of the skeletons of P2 Meflin^−/−^ mice showed accelerated growth of long bones compared with wild-type (WT) littermates (Fig. 7E,F), consistent with the data that Meflin regulated the undifferentiated state of BM-MSCs and C3H10T/1/2 cells and suppressed their differentiation into skeletal lineages (Fig. 5). Further supporting this notion, bone histomorphometric analyses revealed a significant increase in osteoblast number (N.Ob), osteoblast surface (Ob.S) and osteoid surface (OS) per bone surface (BS) and osteoid volume (OV) per bone volume (BV) in the secondary spongiosa area of the tibiae from 10-week-old Meflin^−/−^ mice relative to WT littermates (Fig. 7G). Those data confirmed that Meflin-deficiency directs accelerated differentiation of BMSCs/SSCs into osteoblasts. In addition, quantitative RT-PCR (qPCR) using RNA isolated from the tibiae revealed that the expression of Collagen Ia and Osteocalcin, hallmarks for osteogenic differentiation, was increased in Meflin^−/−^ mice compared with WT mice (Fig. 7H). The differences in osteoclast number (N.Oc) and osteoclast surface (Oc.S) per BS and the thickness of the tibial growth plate (GP.Th) and the GP proliferative zone were not apparent between WT and Meflin^−/−^ mice (Fig. 7G). Those results left the *in vivo* role of Meflin in chondrogenic differentiation unresolved at present.

**Figure 8:**
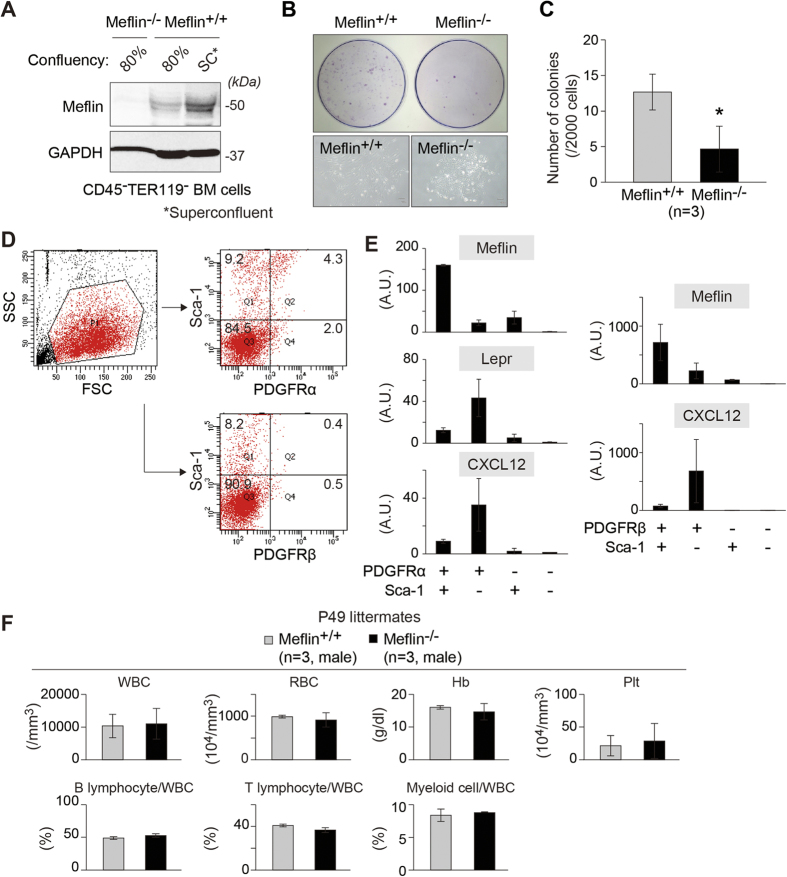
Meflin-deficiency reduced the capacity of non-hematopoietic BM cells to generate CFU-Fs. **(A)** Meflin expression in BM-MSCs isolated from the BM of wild-type and Meflin^−/−^ P56 mice was monitored by Western blot analysis. **(B)** Representative images of CFU-Fs produced from CD45^−^TER119^−^ BM cells from wild-type and Meflin^−/−^ P56 mice stained with the Giemsa solution (top panel). The images for individual representative colonies are also shown (lower panel). **(C)** Lower CFU-F frequency in Meflin^−/−^ CD45^−^TER119^−^ BM cells. The number of CFU-F for 2 × 10^3^ initially plated wild-type and Meflin^−/−^ CD45^−^TER119^−^ BM cells in 10-cm dishes was calculated and quantified (n = 3). *P < 0.05 compared with wild-type mice. **(D)** Representative flow cytometric profile of non-hematopoietic CD45^−^Ter119^−^ BM cells stained for PDGFRα (upper right) and PDGFRβ (lower right) and Sca-1. The cells were isolated from the femurs and tibiae of 8- to 10-week-old mice with collagenase treatment. The percentage of each cell population is shown in the panels. FSC, forward scatter; SSC, side scatter. **(E)** Relative mRNA expression levels of Meflin, *Lepr* and *Cxcl12* was assessed by qPCR for each gated population from CD45^−^Ter119^−^ BM cells. The data for each mRNA were normalized against GAPDH control. **(F)** Quantification of the number of each type of peripheral blood cell from 7-week-old WT and Meflin^−/−^ mice.

Next, we isolated non-hematopoietic CD45^−^Ter119^−^ BM cells from WT and Meflin^−/−^ P56 mice and compared their ability to form CFU-Fs *in vitro* (Fig. 8A–C). We found a significant decrease in the number of clonogenic CFU-Fs derived from Meflin^−/−^ BM cells compared with WT mice. It followed that Meflin^+^ CD45^−^Ter119^−^ BM cells were enriched for CFU-Fs compared to Meflin-negative fractions.

The above finding that Meflin was involved in CFU-F activity prompted us to undertake a detailed analysis for Meflin-expressing BM cells in 8- to 10-week-old mice (Fig. 8D,E). Meflin was most abundantly expressed in a CD45^−^Ter119^−^PDGFRα^+^ Sca-1^+^ (PαS) fraction in the BM that is known to be highly enriched for CFU-F activity^23^. Meflin was also modestly expressed in CD45^−^Ter119^−^PDGFRα^+^ Sca-1^−^ cells, the majority of which represent CAR/Lepr^+^ cells that contain most of the CFU-F activity and function as adipo-osteogenic progenitors in the BM^8^. These data were consistent with the coexpression of Lepr and Meflin in perisinusoidal stromal cells in the BM (Fig. 2D). We also sorted the CD45^−^Ter119^−^ BM cells for the expression of PDGFRβ and Sca-1 and found that PDGFRβ^+^ Sca-1^−^ cells, which also likely represent CAR/Lepr^+^ cells^12^, express modest levels of Meflin (Fig. 8D,E). The data, although preliminary, suggested that Meflin was variably expressed in various types of BM stromal cells, including PαS cells as well as PDGFRα^+^ Sca-1^−^ and PDGFRβ^+^ Sca-1^−^ cells. Moreover, it was likely involved in the self-renewal capacity of those cells in culture, further supporting the view that Meflin was a potential functional marker for MSCs (Fig. S8).

Finally, considering that CAR/Lepr^+^ cells constitute the hematopoietic microenvironment^9^,^10^,^12^,^13^, we investigated the alterations in the hematopoietic system in Meflin^−/−^ mice. Blood cell counts from 7-week- (Fig. 8F) and 6-month- (data not shown) old Meflin^−/−^ mice, however, showed no apparent difference in the number of peripheral blood cells, including red blood cells, platelets, B- and T-lymphocytes and myeloid cells, compared to WT mice. Those results indicated that there was no apparent effect of Meflin-deficiency on the generation of differentiated hematopoietic cells under steady-state conditions. Further studies of peripheral blood cells and BM HSCs at various life stages in mice under different conditions are needed to determine the role of Meflin in the regulation of the hematopoietic microenvironment.

## Discussion

In the present study, we showed that Meflin was selectively expressed in cultured MSCs, pericytes, and fibroblasts, but not other types of cells, where it regulated the undifferentiated state of the cells. We also showed the distribution of Meflin^+^ cells in the BM, adipose tissue, and other organs, where the majority of them were subendothelial or perivascular. These findings suggest that Meflin is a potential marker that defines some populations of cells of origin for MSCs and perivascular cells both *in vitro* and *in vivo*.

Our study has not addressed several issues including *in vivo* functions and the detailed expression pattern of Meflin (Fig. S8). Further studies are needed to clarify in detail which populations of BMSCs/SSCs in the BM and perivascular cells in multiple organs express Meflin to regulate their functions. We are not aware of a functional relationship between Meflin and other markers for BMSCs/SSCs, including Lepr, nestin, CXCL12, SCF, gremlin 1, osterix, CD146, CD105, Mx1, etc.^5^,^9^,^11^. It is presently unclear how Meflin^+^ cells contribute to the remodeling and regeneration of bones and the recovery and regeneration of the HSC niche in the BM (Fig. S8). The skeletal phenotype found in Meflin^−/−^ mice (Fig. 7) hints that Meflin^+^ cells may partially overlap with recently reported osteochondroreticular (OCR) stem cells that fulfill the characteristics of both developmental and postnatal SSCs^24^. However, this possibility requires further validation. It has not yet been addressed whether other pericyte markers (PDGFRβ, α-smooth muscle cell actin, NG2[chondroitin sulfate proteoglycan 4], etc.)^19^ coincide with Meflin in perivascular cells. Also, the involvement of Meflin in the trophic or immunomodulatory activities of cultured MSCs, which have been used in many therapeutic clinical trials^14^,^15^, needs to be clarified.

A significant limitation in our study comes from the absence of a Meflin antibody that can be used for immunofluorescence staining and fluorescence-activated cell sorter analyses. The Meflin antibodies that we have generated, as well as those that are commercially available, are not compatible with those applications. Nonetheless, we believe that the sporadic and perivascular distribution of Meflin^+^ cells in the BM and other organs (Figs 2 and 3), the enriched expression of Meflin in SCF-positive BMSCs and PαS cells in the BM (Figs S4, 8E) and the downregulation of Meflin in trilineage differentiation in cultured MSCs (Fig. 4) indicate the possibility that Meflin defines at least some populations of undifferentiated or immature BMSCs/SSCs and perivascular cells *in vivo*. Supporting this notion, several gene expression studies found that Meflin expression levels ranked among the top 10–50 genes that were highly expressed in cultured BM-MSCs^32^,^33^. Another intriguing finding, although its biological significance has not been assessed, is that Meflin has been frequently detected as an upregulated gene in cancer stroma of breast and pancreatic cancers^50^,^51^ and fibrotic diseases^52^. The changes in Meflin expression that depend on the stiffness of the extracellular substrate (Fig. S6), microgravity^53^, 1,25-dihydroxyvitamin D3 treatment^54^, and senescence^55^ are also intriguing.

Although the mechanism is presently unknown, the regulation of FoxO1 subcellular localization by Meflin disclosed a new aspect of the function of the LIG family of proteins (Fig. 6D,E). It is believed that LIG members interact with RTKs to regulate their downstream signaling pathways^43^, which we found was not the case with Meflin. Given that many of the LIG family members are preferentially expressed in neural tissues to regulate synaptic formation and integrity of neural circuits^56^, it would be important to study whether Meflin interacted with unknown ligands, either on the surface of a juxtaposed cell or with soluble factors, modulating cell-cell communications in the BM and other tissues.

At present, the developmental origin and ontogeny of Meflin^+^ cells remain unclear. In the developing embryo, Meflin expression is segregated in non-neural stromal and skeletal tissues, while its paralogue Linx appears specifically expressed in neural cells (Fig. 2A)^29^,^31^. Recent evidence suggested that BMSCs/SSCs and pericytes originate from both the mesoderm germ layer and neuroectoderm-derived neural crest cells^5^,^57^,^58^. Therefore, it would be tempting to investigate how Meflin^+^ cells and Linx^+^ neurons segregate early from a common progenitor to contribute to the development of non-neural and neural tissues, respectively.

In summary, our study proposes that Meflin could be a novel marker for the precursors of MSCs and their progeny and perivascular cells both *in vitro* and *in vivo*. Development of further immunological tools and reporter mice will help us understand the biological significance of Meflin in the biology of MSCs and perivascular cells.

## Methods

### Cell culture and differentiation

3T3-L1, NIH3T3 and C3H10T1/2 cells were purchased from the American Type Culture Collection (ATCC) and were cultured in 3T3-L1 Preadipocyte Medium (ZenBio), the equivalent of Dulbecco’s modified Eagle’s medium (DMEM) supplemented with 10% fetal bovine serum (FBS). COS7 (ATCC), 293FT (Clonetech), and Flip-In 293 (Invitrogen) cells were cultured in DMEM (Nakalai Tesque, Kyoto, Japan) supplemented with glucose and 10% FBS. Human dermal fibroblasts (NHDF-Ad), BM-MSCs, and ADSCs were purchased from Lonza and cultured in FGM-2, MSCGM and ADSC media (Lonza), respectively. Other primary cells used in the study are described in Supplementary Information. For trilineage differentiation of C3H10T1/2 and BM-MSCs, cells were grown to be superconfluent on culture dishes, followed by the induction of differentiation by hMSC Osteogenic (PT-3002), Chondrogenic (PT-3003) or Adipogenic (PT-3004) Differentiation Medium BulletKit (Lonza). For adipogenic differentiation of confluent 3T3-L1 cells, cultures were incubated for an additional 48 h, followed by the induction of differentiation by 3T3-L1 Differentiation Medium and Adipocyte Medium (ZenBio).

### Histology, cell biology, biochemistry and flow cytometry

Detailed protocols for ISH, gene expression analysis and cell biological and biochemical experiments are described in Supplementary Information.

### Meflin-deficient mice

Targeted embryonic stem (ES) cell clones (EPD0788_2_H09, EPD0788_2_B12) isolated from JM8 cells (C57BL/6N background) were purchased from the European Mouse Mutant Cell Repository (EuMMCR). Quality control for correct targeting and homologous recombination in the 5′ and 3′ homology arms was assessed by long range PCR by the EuMMCR. The Meflin*/islr* gene consisted of three exons (an exon 1, an alternative exon 1, and exon 2), where exon 2 contained the whole open reading frame (ORF). The targeting construct was designed by the EuMMCR to flank the alternative exon 1 and the whole Meflin ORF (exon 2) with loxP sites and later delete them together with the neo-cassette via Cre recombinase (Fig. 7A). The targeted ES cells were injected by Institute of Immunology Co. Ltd. (Tokyo, Japan) into Balb/c blastocysts and the resulting chimeric male mice were mated with C57BL/6J female mice to generate F1 animals that were heterozygous for the Meflin floxed allele, which were then crossed with actin-Cre transgenic mice to obtain a null allele. All animal protocols were approved by the Animal Care and Use Committee of Nagoya University Graduate School of Medicine. All the *in vivo* experiments were performed in compliance with Nagoya University’s Animal Facility regulations.

## Additional Information

**How to cite this article**: Maeda, K. *et al*. Identification of Meflin as a Potential Marker for Mesenchymal Stromal Cells. *Sci. Rep*. **6**, 22288; doi: 10.1038/srep22288 (2016).

## Supplementary Material

Supplementary Information
