# Supplementary material for: Identification of Meflin as a Potential Marker for Mesenchymal Stromal Cells
Source: Sci Rep. 2016 Feb 29;6:22288. doi: 10.1038/srep22288 (PMC4770287; doi:10.1038/srep22288)
Supplement: Supplementary Information [file srep22288-s1.pdf]

Identification of Meflin as a Potential Marker for Mesenchymal Stromal Cells

Keiko Maeda, Atsushi Enomoto, Akitoshi Hara, Naoya Asai, Takeshi Kobayashi, Asuka Horinouchi, Shoichi Maruyama, Yuichi Ishikawa, Takahiro Nishiyama, Hitoshi Kiyoi, Takuya Kato, Kenju Ando, Liang Weng, Shinji Mii, Masato Asai, Yasuyuki Mizutani, Osamu Watanabe, Yoshiki Hirooka, Hidemi Goto and Masahide Takahashi

Supplementary Information

1. Supplementary Table 1 and Figures 1-8
2. Supplementary Methods
3. Supplementary References

Supplementary Table 1. A list of genes up-regulated (log2 ratio > 4) at Day 3 compared with Day 0 in both 3T3-L1 and NIH3T3 cells but not HT-1080 fibrosarcoma cells

	3T3-L1			NIH3T3			HT-1080		
	Day 3/Day 0 (log2 ratio)	Day 3 gScale signal	Day 0 gScale signal	Day 3/Day 0 (log2 ratio)	Day 3 gScale signal	Day 0 gScale signal	Day 3/Day 0 (log2 ratio)	Day 3 gScale signal	Day 0 gScale signal
lumican (*Lum*)	8.95	2055.5	4.1	6.54	125137.1	1349.1	0.20	6.3	5.4
insulin-like growth factor 2 (*Igf2*), transcript variant 1	8.32	1638.8	5.1	6.48	46772.6	524.9	-1.03	6.6	13.4
immunoglobulin superfamily containing leucine-rich repeat (*Islr*)	7.84	6862.3	30.0	5.09	4177.4	122.3	0.38	140.2	107.7
apolipoprotein D (*Apod*)	6.94	10666.5	86.9	8.21	1361.6	4.6	0.15	6.9	6.2
microfibrillar-associated protein 4 (*Mfap4*)	6.57	12033.1	126.3	8.86	11628.5	25.0	0.19	6.1	5.4
decorin (*Dcn*)	6.16	94465.6	1323.7	4.92	409097.0	13491.8	-3.32	5.2	52.2
2'-5' oligoadenylate synthetase-like 2 (*Oasl2*)	6.15	49920.7	704.5	4.38	89.3	4.3	-0.30*	241.4	296.3
avian musculoaponeurotic fibrosarcoma (v-maf) AS42 oncogene homolog (*Maf*)	5.42	661.8	15.4	4.68	497.7	19.4	2.55	917.5	156.6
ATP-binding cassette, sub-family A (ABC1), member 1 (*Abca1*)	5.39	560.1	13.3	6.07	293.7	4.4	2.86	2237.3	308.5
melan-A (*Mlana*)	5.38	788.6	19.0	5.63	1610.8	32.6	-0.51	12.3	17.5
retinoic acid receptor responder (tazarotene induced) 2 (*Rarres2*)	5.22	2733.2	73.2	5.20	1109.8	30.2	0.19	5.4	4.7
dermatopontin (*Dpt*)	5.17	5538.2	154.1	7.46	6964.6	39.6	0.49	190.8	135.6
calcium channel, voltage-dependent, T type, alpha 1G subunit (*Cacna1g*)	5.12	3711.9	106.5	4.09	2502.6	147.1	0.38	47.4	36.5
tripartite motif-containing 63 (*Trim63*)	5.05	144.1	4.4	4.14	603.8	34.3	3.11	242.7	28.0
complement component 3 (*C3*)	5.03	39143.1	1198.2	6.74	24646.4	231.3	3.25	716.3	75.5
angiotensin II receptor, type 2 (*Agtr2*)	4.86	151.5	5.2	10.13	6172.2	5.5	0.19	7.6	6.7
insulin-like growth factor binding protein 5 (*Igfbp5*)	4.81	134.5	4.8	4.59	93.2	3.9	0.18	6.4	5.6
carboxypeptidase A2, pancreatic (*Cpa2*)	4.65	238.3	9.5	4.07	110.0	6.5	0.24	6.0	5.1
multiple EGF-like-domains 6, transcript variant 1 (*Megf6*)	4.60	157.0	6.5	4.25	66.9	3.5	-0.77	142.6	243.7
glycerophosphodiester phosphodiesterase domain containing 2 (*Gdpd2*)	4.57	777.6	32.8	4.40	2710.7	128.3	0.19	6.3	5.5
FXYD domain-containing ion transport regulator 1 (*Fxyd1*), transcript variant 1	4.53	114.3	5.0	6.82	754.4	6.7	-1.34	6.6	16.7
complement component 1, s subcomponent (*C1s*), transcript variant 1	4.50	3773.8	166.5	4.03	18280.5	1119.2	2.07	7347.5	1754.0
protein phosphatase 1, regulatory (inhibitor) subunit 3C (*Ppp1r3c*)	4.47	789.4	35.7	4.04	64.0	3.9	-0.28	105.1	127.5
H19 fetal liver mRNA (*H19*)	4.46	168.4	7.6	5.89	1937.8	32.7	0.03**	101646.7	99824.2
Zinc finger RNA binding protein 2 (*Zfr2*)	4.34	152.4	7.5	4.24	127.6	6.8	-0.09	21.4	22.8
osteoglycin (*Ogn*)	4.28	18536.3	955.3	4.18	50693.1	2797.9	0.18	7.6	6.7
alcohol dehydrogenase 1 (class I) (*Adh1*)	4.27	216.0	11.2	5.87	1458.7	24.9	0.17	7.2	6.4
asporin (*Aspn*)	4.19	3518.5	192.9	6.68	4060.3	39.7	0.17	5.2	4.6
Spi-B transcription factor (Spi-1/PU.1 related) (*Spib*)	4.18	224.2	12.4	5.69	241.7	4.7	-0.45	28.3	38.7
periostin, osteoblast specific factor (*Postn*)	4.14	66107.4	3747.9	4.56	84817.2	3603.8	0.19	5.4	4.8
nidogen 2 (*Nid2*)	4.04	6738.2	410.8	4.33	18856.4	940.5	2.11	1253.3	289.5

*Homo sapiens 2'-5'-oligoadenylate synthetase-like (*OASL*), transcript variant 1, mRNA [NM_003733]

**Homo sapiens testis enhanced gene transcript (BAX inhibitor 1) (*TEGT*), transcript variant 1, mRNA [NM_003217]

```
                    Signal peptides      Leucine-rich repeat N-terminal domain           LRR
Meflin (Islr)  MRALCLLCWA-VLLNLVRACPEPCDCGEKYGFQIADCAYRDLEGVPPGFPANVTTLSLSA
 Linx (Islr2)  MGPFGALCLAWALLGVVRACPEPCACVDKYAHQFADCAYKELREVPEGLPANVTTLSLSA
               * .:  ** * .**.:******** * :**..*:*****::*. ** *:***********

                                         LRR                        LRR
               NRLPGLPEGAFREVPLLQSLWLAHNEIRSVAIGALAPLSHLKSLDLSHNLLSEFAWSDLH
               NKITVLRRGAFVNVTQVTSLWLAHSEVRTVESGALAVLSQLKNLDLSHNLISNFPWSDLR
               *::. * .*** :*. : ******.*:*:*  **** **:**.*******:*:*.****:

                             LRR                         LRR
               NLSALQLLKMDSNELAFIPRDAFSSLSALRSLQLNHNRLHALAEGTFAPLTALSHLQIND
               NLSALQLLKMNHNRLGSLPRDALGALPDLRSLRINNNRLRTLEPGTFDALSALSHLQLYH
               **********: *.*. :****:.:*. ****::*:***::*  *** .*:******: .

                         Leucine-rich repeat C-terminal domain
               NPFDCTCGIVWFKTWALASAVSIPEQDNIACTTPHVLKGIPLGRLPPLPCSAPSVQLSYQ
               NPFHCSCGLVWLQAWAASTRVSLPEPDSIACASPPELQGVPVHRLPALPCAPPSVRLSAE
               ***.*:**:**:::** :: **:** *.***::*  *:*:*: ***.***:.***:** :

                                         Immunoglobulin C-2 Type
               PS--QDGAELRPGFVLALHCDVDGQPVPQLHWHIHTPGGTVEIASPNVG--TDG------
               PPPEAPGTPLRAGLAFMLHCVAEGHPTPRLQWQLQIPGGTVVLVPPVLSKEEDGGDKVED
               *.    *: **.*:.: *** .:*:*.*:*:*::: ***** :..* :.   **

               ------------------RALPGALATSGQPRFQAFANGSLLIPDFGKLEEGTYSCLATN
               GEGDGDEDLPTQTEAPTPTPAPAWPAPPATPRFLALANGSLLVPLLSAKEAGIYTCRAHN
                                  . *.  *... *** *:******:* :.  * * *:* * *

               ELGSAESSVNVALATPG-------------------------------------EG----
               ELGTNSTSLRVTVAAAGPPKHAPGTGEEPDAQVPTSERKATTKGRSNSVLPFKPEGKTKG
               ***: .:*:.*::*:.*                                     **

               ---------------------GE--------DAVGHKFHGKAVEG----KGCYTVDNEVQ
               QGLARVSVLGEIEAELEETDEGEQMEGQIPADPMGEKHCGHGDPSRYVSNHAFNQSSDLK
                                    **        *.:*.*. *:.  .    : .:. ..:::

               P-----------------------------SGPEDN----------VVIIYLSRAG---P
               PHVFELGVIALDVAEREARVQLTPLAARWGPGPDGASGARRPGRRPLRLLYLCPAGGGTA
               *                             .**:.           : ::**. **   .
             Potential GPI-modification site
                      ↓
               PEAAIAADG---------RPAQQFSGILLLG----------------QSLLVLSFFYF--
               VQWSRVEEGVNAYWFRGLRPGTNYSVCLALAGEACHVQVVFSTKKELPSLLVIVTVSVFL
                : : . :*         **. ::*  * *.                 ****:  . .

               ------------------------------------------------------------
               LVLATVPLLGAACCHLLAKHPGKPYRLILRPQAPDPMEKRIAADFDPRASYLESEKSYPA
             Transmembrane domain

               ------------------------------------------------------------
               RGEAGGEEPEEVPEEGLDEDVEQGDPSGDLQREESLAGCSLVESQSKANQEEFEAGSEYS

               -------------------------
               DRLPLGAEAVNIAQEINGNYRQTAG
```

Figure S1. Sequence alignment of amino acid for mouse Meflin (Islr) and Linx (Islr2)
Each protein has a secretion signal peptide at the N-terminus, a leucine-rich repeat N-terminal domain, five leucine-rich repeat (LRR) domains, a leucine-rich repeat C-terminal domain, and an immunoglobulin-like domain. Meflin has a potential GPI-modification site at the C-terminus (arrow), whereas Linx has a transmembrane domain (pink region) and a cytoplasmic region.

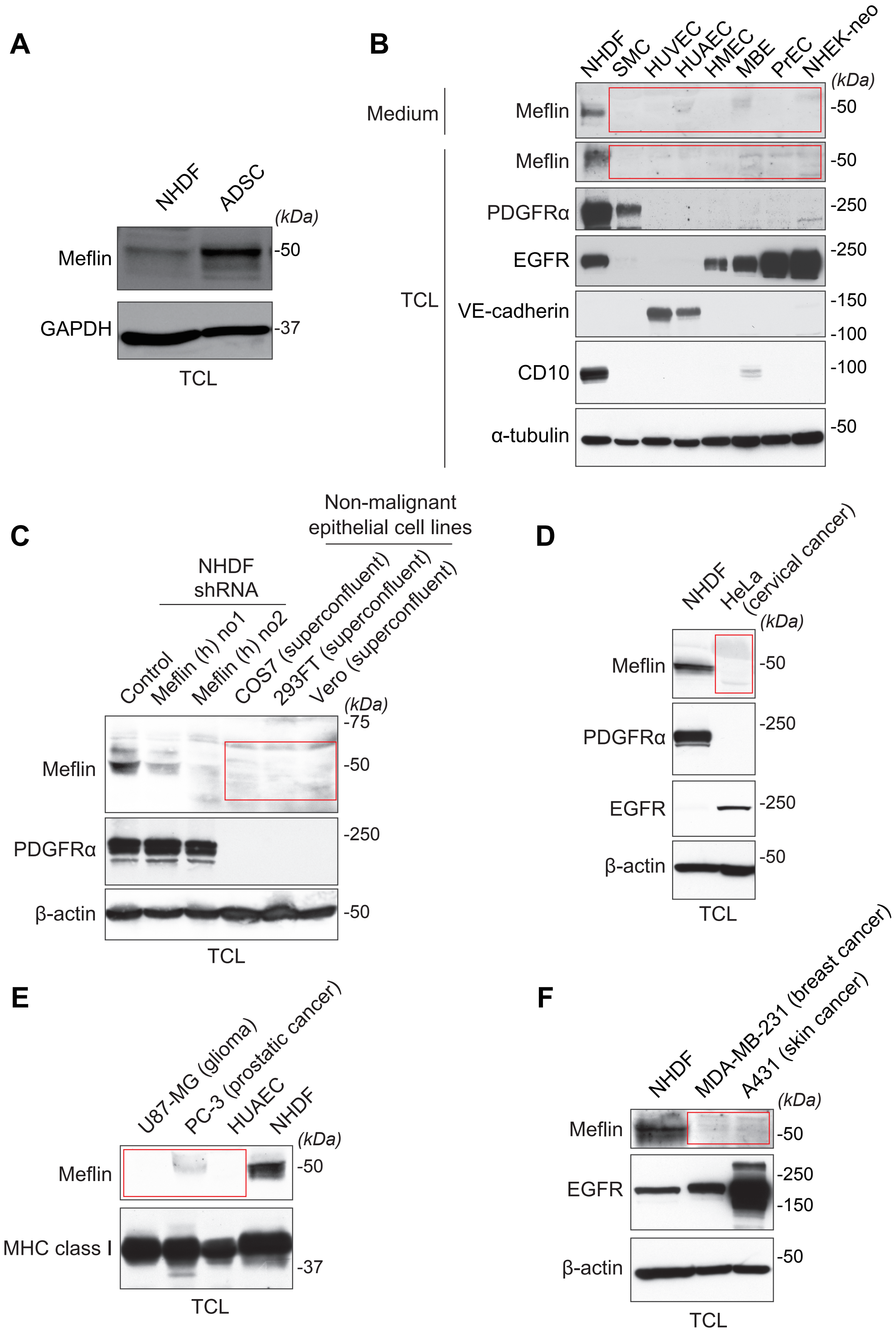

Figure S2. Meflin expression in primary cultured cells and immortalized cell lines

(A) Meflin expression in NHDF (dermal fibroblasts) and adipose tissue-derived stem cells (ADSCs). TCL, total cell lysates.

(B) No apparent expression (red boxes) of Meflin protein in smooth muscle, aortic and venous endothelium or breast, prostatic, and skin epithelial cells. Lysates from the indicated cells were examined by Western blot analysis. NHDF served as a positive control. SMC, aortic smooth muscle cells; HUVEC, human umbilical cord endothelial cells; HUAEC, human aortic endothelial cells; HMEC, human mammary epithelial cells (Lonza); MBE, human mammary basal epithelial cells (ZenBio); PrEC, human prostatic epithelial cells; NHEK-neo, human skin keratinocytes.

(C-F) No apparent expression (red boxes) of Meflin in immortalized non-malignant cell lines or cancer cell lines.

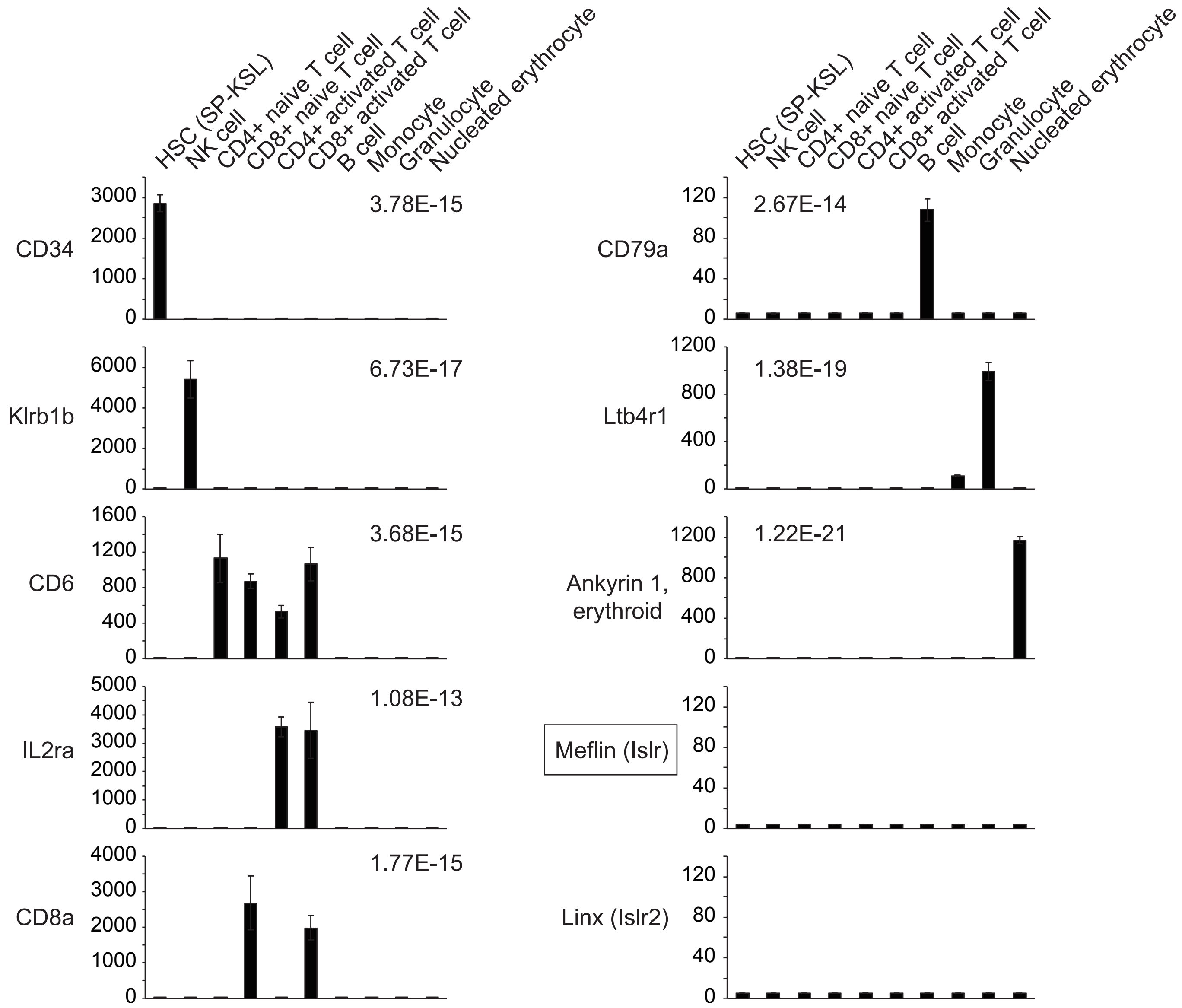

Figure S3. No expression of Meflin mRNA in hematopoietic stem cells (HSCs) or their differentiated progeny
The data were extracted from a previous study by the Goodell laboratory[1] and its accompanying microarray dataset (GSE6506) and illustrated graphically. See Chambers et al. for the detailed protocol to selectively isolate mouse HSCs and cells of each lineage. Note that neither Meflin (*Islr*) nor Linx (*Islr2*) expression was detected in any of the cells tested.

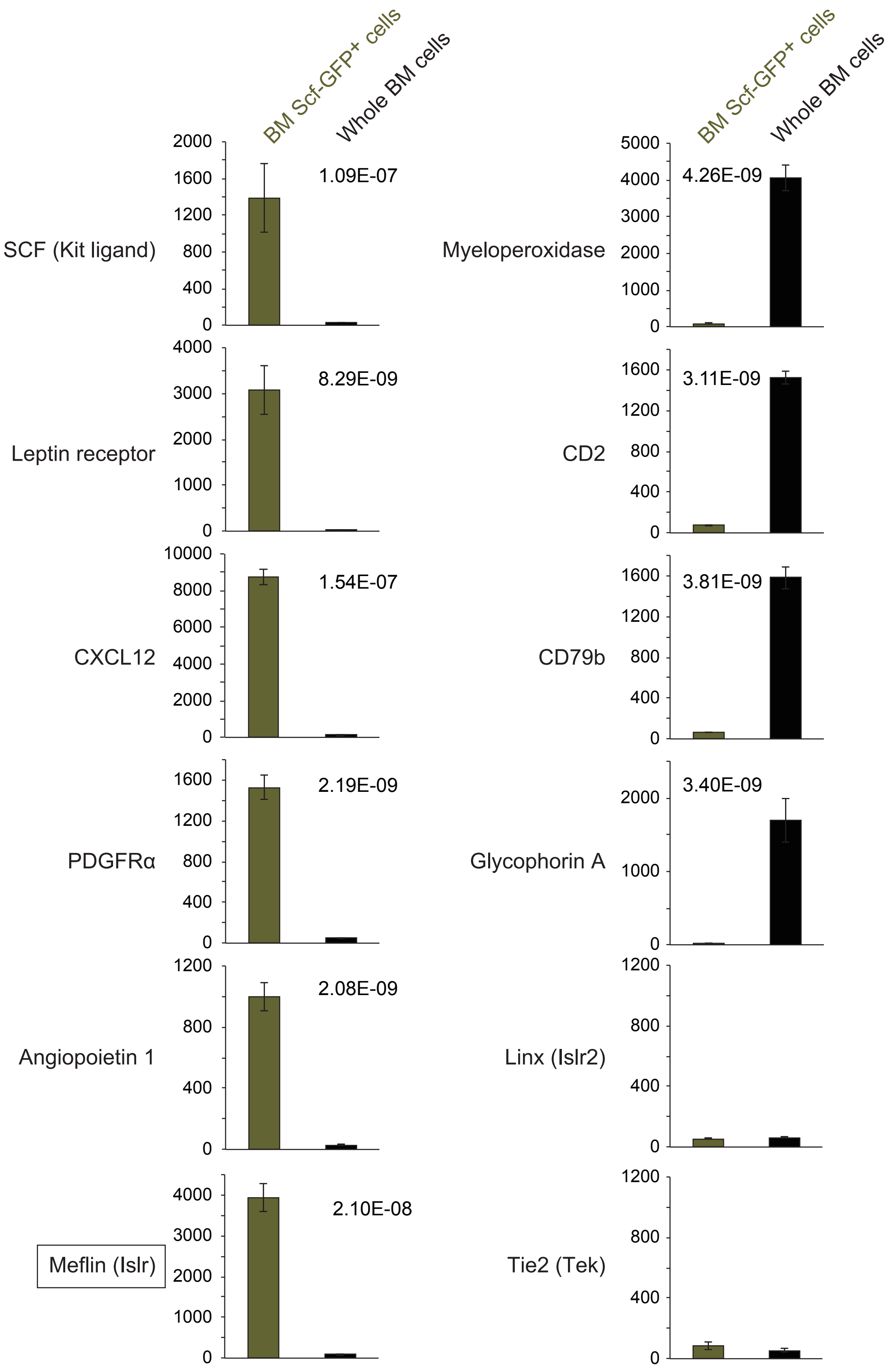

Figure S4. Meflin gene expression was elevated in cells enriched for the leptin receptor and CXCL12 in SCF-positive BMSCs compared to whole BM cells

The data were extracted from a previous study by the Morrison laboratory[2] and its accompanying microarray dataset (GSE33158) and illustrated graphically. Meflin (*Islr*) expression was enriched along with that of SCF, leptin receptor, CXCL12, PDGFRα and angiopoietin 1 in SCF-GFP$^+$ cells isolated from the BM of SCF-GFP knock-in mice. CD2-, CD79-, glycophorin A-, and myeloperoxidase-positive hematopoietic lineage cells were present in whole BM cells. Note that Linx (*Islr2*), a paralogue of Meflin, and *Tie2*, an endothelial marker, were not detected in SCF-GFP$^+$ cells at levels above the background found in whole BM cells.

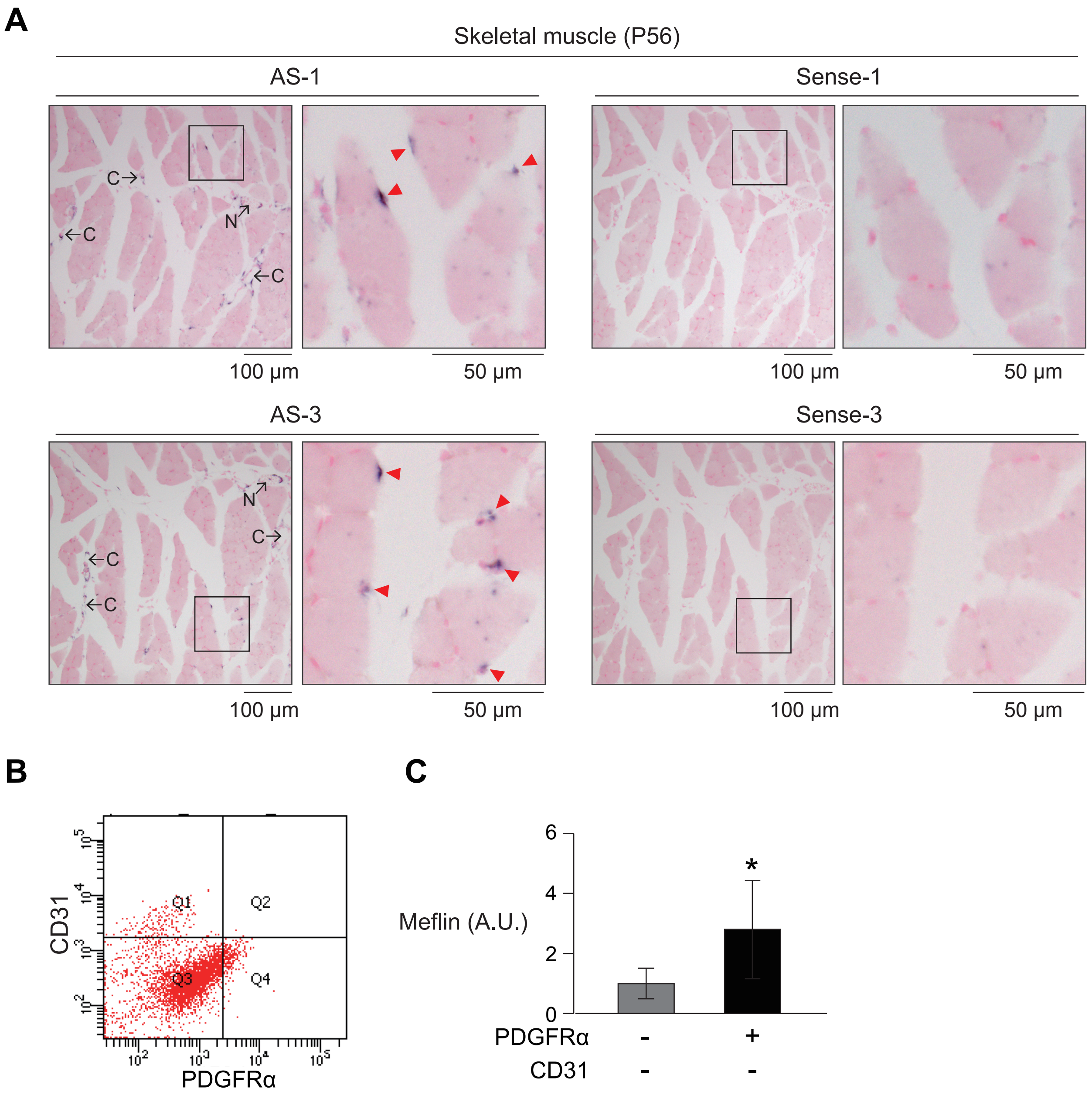

Figure S5. Meflin expression in the skeletal muscle

(A) ISH analysis of the skeletal muscle of P56 C57BL/6 mice with Meflin antisense (AS-1, AS-3) and control (Sense-1, Sense-3) probes. Box regions are magnified in adjacent panels. Red arrowheads indicate Meflin^{+} cells were located at the edges of muscle fibers. Note that Meflin^{+} cells were also observed in pericytes and perivascular fibroblasts around the capillaries (C) and perineurium cells around the nerve (N).

(B) Representative flow cytometric profile of non-hematopoietic CD45^{-}Ter119^{-} cells stained with PDGFRα and CD31 that were isolated from hind limb muscles of 8- to 10-week-old mice with collagenase treatment.

(C) Relative mRNA expression levels of Meflin were assessed by qPCR for each population from muscle-derived CD45^{-}Ter119^{-} cells. An asterisk indicates a statistically significant difference ($P < 0.05$).

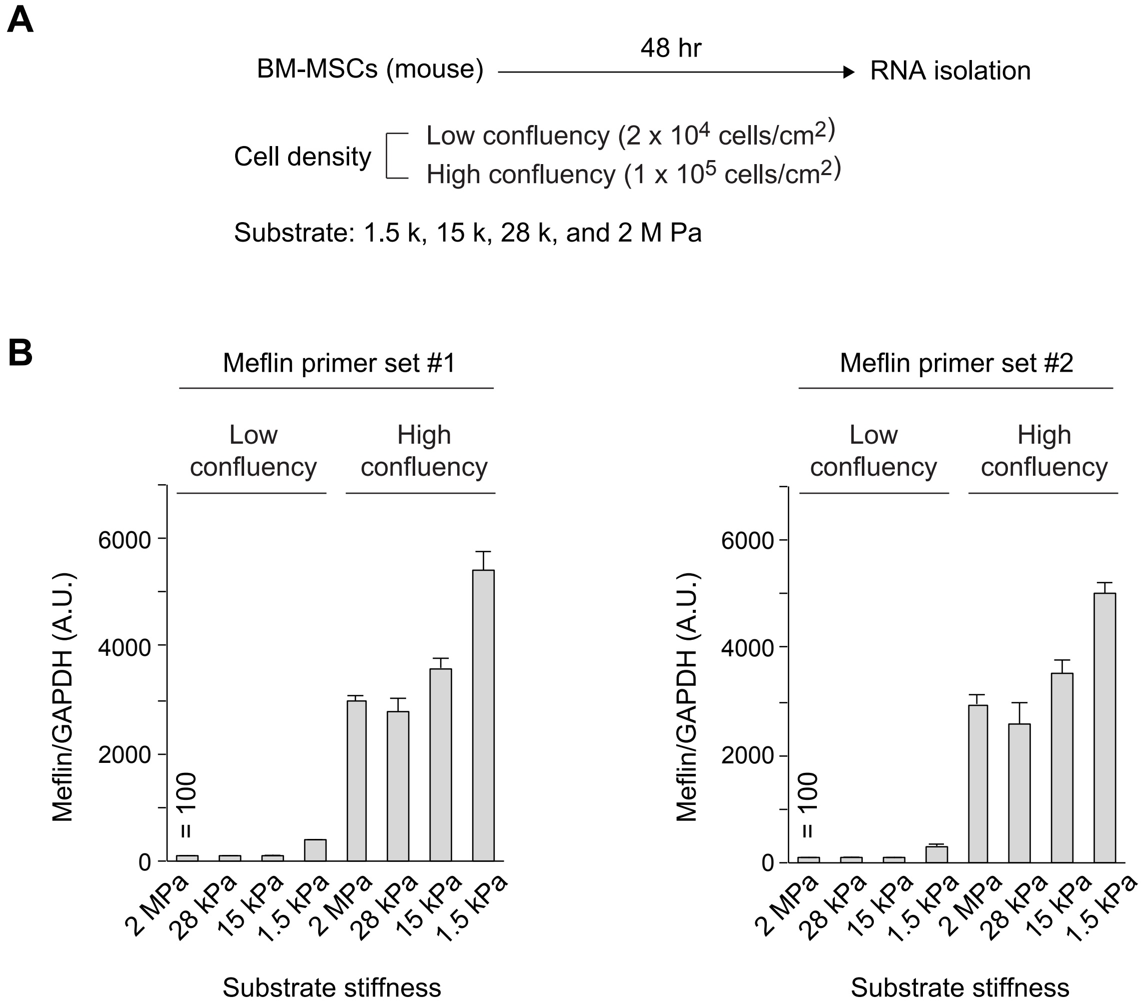

Figure S6. Meflin expression depended on substrate stiffness in cultured MSCs

(A) Mouse BM-MSCs were seeded on a substrate with the indicated stiffness at either low or high confluency.

(B) The expression of Meflin was measured by qPCR and normalized to *Gapdh*. Independent primer sets (set #1 and #2) were used to confirm the reproducibility. Note that Meflin expression was detected only in BM-MSCs cultured at high confluency, which largely depended on substrate stiffness.

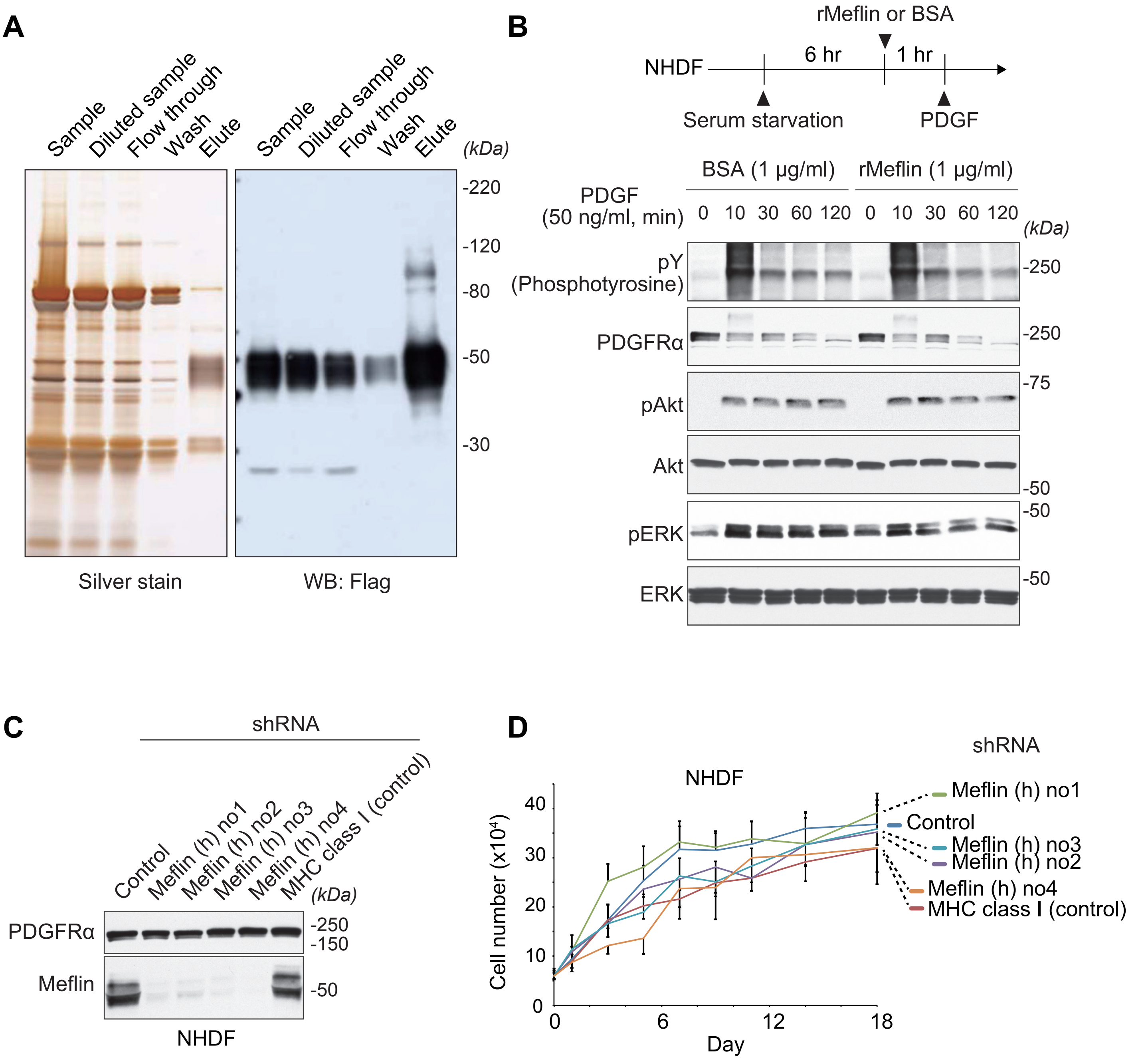

Figure S7. No apparent effect of the secreted form of Meflin on PDGF signaling in fibroblasts
(A) Production and purification of recombinant Meflin by the silkworm expression system. The purity of the final eluate was calculated to be approximately 76% based on a densitometric assessment of the silver staining.
(B) Recombinant Meflin (rMeflin) and control bovine serum albumin (BSA) were added to fibroblasts (NHDFs) starved in serum-free medium to achieve the final concentration of 1 μg/mL. After one h, the cells were stimulated by recombinant rat PDGF-BB (50 ng/mL), followed by Western blot analysis with the indicated antibodies. No apparent effect of rMeflin on PDGF signaling was observed when the activation of Akt or ERK was used as readout.
(C, D) No apparent role of Meflin in cell proliferation. The numbers of NHDFs, which were transduced with retroviruses harboring control or four independent Meflin-specific shRNAs **(C)**, were counted every two days. There was not a clear trend in cell proliferation among the groups, despite variability in the early proliferative phase **(D)**.

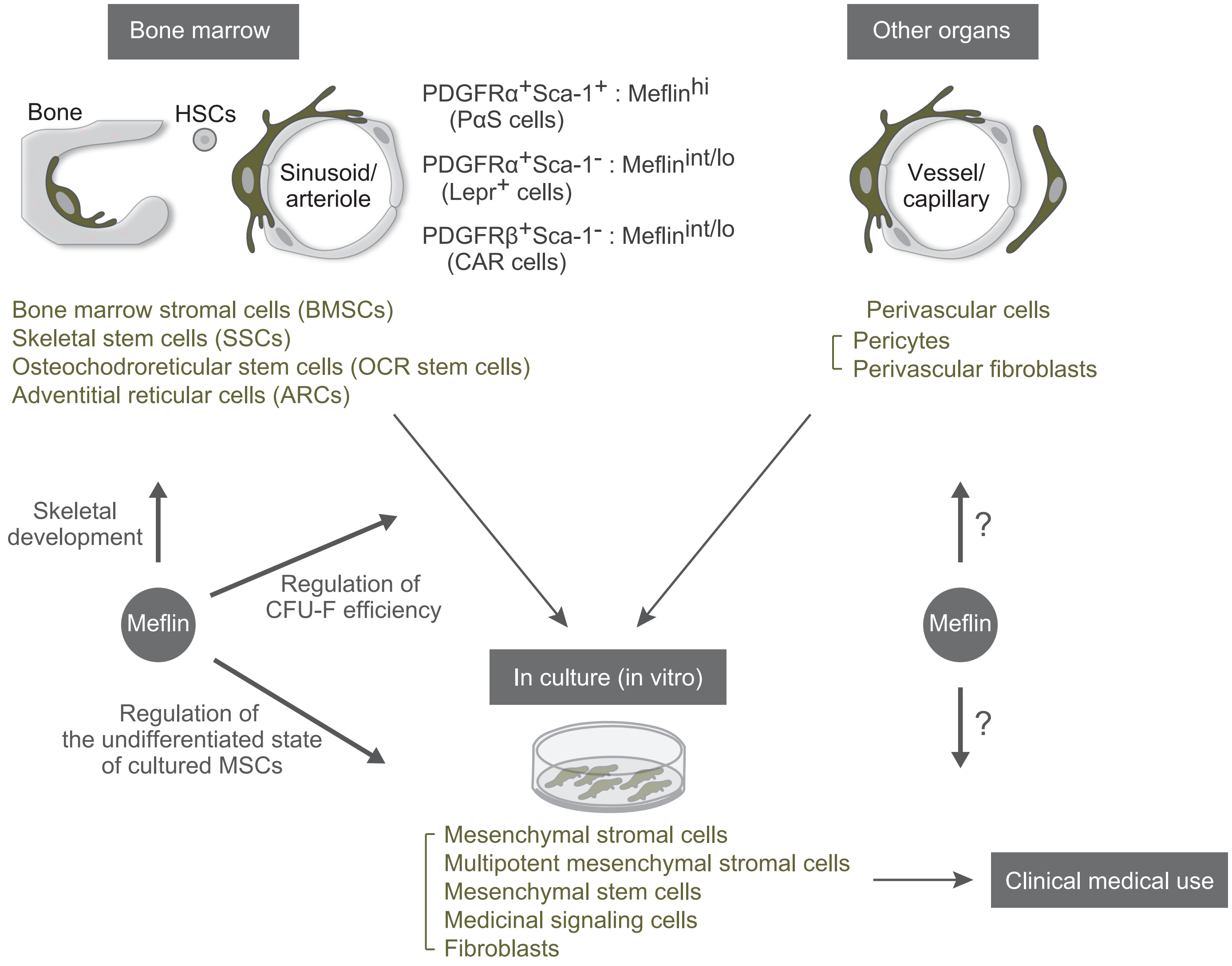

Figure S8. Summary and limitations of the present study

The characteristic distribution pattern of Meflin$^+$ cells in the BM and other organs, when combined with the biochemical data on cultured MSCs, leads to the hypothesis that Meflin is a potential marker for MSCs and pericyte/perivascular fibroblasts both *in vitro* and *in vivo*. There are, however, many questions that must be addressed in future studies. The first challenge is to develop antibodies or tools to prospectively isolate Meflin$^+$ cells from the BM and other sources. Second, a new concept posits that bone marrow stromal cells (BMSCs) are skeletal stem cells (SSCs) and that those cells partially overlap but are not identical with other stromal cells in the BM. It has been suggested that those cells are self-renewing and also serve as a niche for hematopoietic stem cells (HSCs)[3-6]. Given those hypotheses, it will be important to determine whether Meflin is involved in the aforementioned processes as well as the functions of BMSCs/SSCs. Recent studies have suggested that SSCs in the BM consist of distinct populations of cells that are located around the trabeculae and have important functions for bone development and postnatal regeneration. Moreover, they are found around the perisinusoidal region and are essential for bone remodeling and regeneration in postnatal life[7]. At present, a role for Meflin in bone remodeling in postnatal life is unknown. Third, the overlap between Meflin and many other markers for BMSCs/SSCs (leptin receptor, nestin, CXCL12, gremlin 1, SCF, osterix, NGFR/CD271, MCAM/CD146, Stro-1, Mx1, etc.) is another major concern. Fourth, the relevance of Meflin in the biology of pericytes and perivascular fibroblasts remains unknown. For example, the relevance of Meflin in vascular maturation and integrity was not investigated in the present study. Finally, the involvement of Meflin in trophic effects or immunomodulatory functions of cultured MSCs, which have been used in therapeutic clinical trials (http://www/FDA.gov), needs to be clarified[8].

Supplementary Methods

DNA microarray analysis

3T3-L1, NIH3T3 and HT-1080 cells were cultured to the indicated degree of confluency. Total RNA was extracted with RNeasy Plus Mini Kits (Qiagen). Cyanine-3 (Cy3)-labeled cRNA was prepared from 500 ng RNA using the Quick Amp Labeling Kit (Agilent) according to the manufacturer's instructions. Fragmentation of Cy3-labelled cRNA and hybridization to an Agilent Whole Mouse Genome Microarray 4 x 44K were performed by Dragon Genomics Center (Takara Bio, Yokkaichi, Japan). The data have been deposited in Gene Expression Omnibus (GEO; accession numbers GSE69320 and GSE69534).

Antibodies

Rabbit anti-mouse and anti-human Meflin polyclonal antibodies were developed against the keyhole limpet hemocyanin-conjugated 19 (NVALATPGEGGEDAVGHKF) and 23 (CSAPSVQLSYQPSQDGAELRPGF) amino acid sequences of mouse and human Meflin proteins, respectively. The antisera were purified as bound fractions from peptide-conjugated columns. We also generated guinea pig anti-Meflin polyclonal antibody against 25 amino acid (LATPGEGGEDTLGRRFHGKAVEGKG) sequences of human Meflin. Other antibodies used in the study are described in Supplemental Information.

Other antibodies used in the study included monoclonal anti-β-actin (Sigma), polyclonal anti-glyceraldehyde-3-phosphate dehydrogenase (GAPDH) (Abcam), polyclonal anti-Sox9 (Millipore), monoclonal anti-histone-H3 (Millipore), polyclonal anti-Flag (Sigma), monoclonal anti-T7 (Novagen), monoclonal anti-CD59 (Santa Cruz) and polyclonal anti-MHC class I (Epitomics). The following were obtained from R&D Systems: polyclonal anti-collagen II, polyclonal anti-FABP4, polyclonal anti-osteopontin and polyclonal anti-leptin receptor. The following were obtained from Cell Signaling Technology: monoclonal anti-Runx2, monoclonal anti-Foxo1, polyclonal anti-phospho-Foxo1/Foxo3 (Thr24/Thr32), polyclonal anti-PDGFRα, polyclonal anti-EGFR, monoclonal anti-PPARγ, monoclonal anti-Akt, polyclonal anti-phospho-Akt, polyclonal anti-ERK and polyclonal anti-phospho-ERK.

Primary cultured cells

Most of the primary cells used in the study were purchased from Lonza, including human aortic smooth muscle cells (SMC), human umbilical venous endothelial cells (HUVEC), human umbilical arterial endothelial cells (HUAEC), human mammary epithelial cells (HMEC), normal primary prostate epithelial cells (PrEC) and neonatal normal human epidermal keratinocytes (NHEK-neo).

We also used human mammary basal epithelial cells (MBE, ZenBio) and human pericytes from placenta (hPC-PL, PromoCell).

RNA interference

Target sequences for shRNA-mediated knockdown of human (NM_005545.3) and mouse (NM_012043.4) Meflin were designed and generated by iGENE Therapeutics (Tsukuba, Japan) as follows (h and m indicate human and mouse, respectively): Meflin (h) no. 1, 5'-GCCAAAGCCTCCTCCTCTT -3' (nucleotides 1529–1547); Meflin (h) no. 2, 5'-AGATCAACGAGAACCCCTT -3' (nucleotides 809–827); Meflin (h) no. 3, 5'-GACTAGGATAGAATTTGAT -3' (nucleotides 1752–1770); Meflin (h) no. 4, 5'-AACTCAACCACAACCGCTT -3' (nucleotides 737–755); Meflin (m) no. 1, 5'-CAGGGCAACAACAATGTCTTA -3' (nucleotides 1858–1878); Meflin (m) no. 2, 5'-GGCACAAGTTCCATGGCAA -3' (nucleotides 1335–1353); Meflin (m) no. 3, 5'-AGGGCAACAACAATGTCTT -3' (nucleotides 1859–1877). A control sequence provided by Clonetech or a sequence targeted for human MHC class I (5'- GCTACTACAACCAGAGCGAG -3')[9] and firefly luciferase (5'- GTGCGCTGCTGGTGCCAAC -3'; designed by iGENE Therapeutics) was used for negative controls. The oligonucleotide pair was annealed and inserted into the pSIREN-RetroQ retroviral shRNA expression vector (Clonetech). To produce retroviral supernatants, GP2-293 packaging cells (Clonetech) were seeded in collagen type I-coated 100-mm cell culture dishes and transfected with the pVSV-G (vesicular stomatitis virus G protein) vector and either control or Meflin (h/m) shRNA-containing pSIREN-RetroQ vector using Lipofectamine 2000 reagent (Invitrogen). The medium was replaced 24 h later, and virus-containing supernatants were harvested 48 h post-transfection and used for following infection of NHDF-Ads, C3H10T1/2, and 3T3-L1 cells.

Expression of Meflin by the retroviral expression system

The cDNA encoding Meflin was inserted into the pRetroQ retroviral expression vector (Clonetech), followed by production of recombinant retrovirus and infection to C3H10T1/2 cells as described previously[10].

Western blot analysis and immunoprecipitation

For Western blot analysis, cells were lysed with SDS sample buffer (10 mM Tris-HCl, 2% SDS, 2 mM EDTA, 0.02% bromophenol blue, 6% glycerol, pH 6.8), reduced with 80 mM dithiothreitol for 2 h at 50ºC and separated by SDS-polyacrylamide gel electrophoresis (PAGE). Proteins were

transferred to nitrocellulose membranes, blocked in 4% milk in phosphate-buffered saline (PBS) containing 0.05% Tween 20, incubated with primary antibodies and detected by horseradish peroxidase-conjugated secondary antibodies (Dako). In Western blot analysis for the detection of Meflin, the primary antibodies were diluted with Can-Get-Signal Solution 1 (Toyobo, Osaka, Japan) to enhance antibody-antigen binding. For immunoprecipitation analysis, 293FT or COS7 cells were lysed in a buffer containing 20 mM Tris-HCl (pH 7.4), 120 mM NaCl, 1 mM EDTA, and 1% Triton X-100 supplemented with Complete Protease Inhibitor cocktail (Roche) and PhosSTOP phosphatase inhibitor cocktail (Roche, Indianapolis, Indiana). Lysates were cleared by centrifugation at 12,000 x g for ten min, followed by immunoprecipitation using the indicated antibodies and protein A/G beads (Sigma).

Plasmids and stable cell lines

Mouse Meflin/Islr cDNA obtained from Open Biosystems (clone ID. 3966024) was subcloned into a mammalian expression vector pcDNA3.1D-V5-His-TOPO (Invitrogen) and a retroviral expression vector pRetroQ (Clonetech). cDNA encoding mouse PDGFRα was purchased from Open Biosystems (clone ID. 5704645). It was subcloned into a mammalian expression vector pcDNA3.1D-V5-His-TOPO (Invitrogen). Plasmids encoding the ErbB family members and Decorin were generously provided by Shigeki Higashiyama (Ehime University) and Akihiko Matsumine (Mie University), respectively.

For the construction of plasmids encoding SP-T7- and SP-Flag-Meflin, the epitope tags with a secretion SP (21-mer) derived from the Igk-chain were added at the N-terminus of mouse Meflin (amino acid residues 19-428). For generation of 293 cells stably expressing Meflin by flippase (Flp) recombinase-mediated integration, Meflin cDNA subcloned into the pcDNA5/FRT vector (Invitrogen) was cotransfected with pOG44 vector (Invitrogen) into Flp-In 293 cells by Lipofectamine 2000 (Invitrogen), followed by selection for hygromycin (180 μg/mL).

Immunofluorescent staining

Flp-In 293 cells stably expressing Meflin were grown on poly-d-lysine (PDL)-coated glass base dishes, fixed in 4% (w/v) paraformaldehyde, permeabilized with PBS containing 0.05% (v/v) TritonX-100 and then incubated with the anti-Meflin antibody, followed by staining with Alexa 488-conjugated goat anti-mouse or anti-rabbit IgG (Invitrogen). After washing in PBS, fluorescence was visualized with a confocal laser scanning microscope (LSM 700, Carl Zeiss).

Isolation of GPI-anchored proteins and cell surface and nuclear proteins

Triton X-114 phase separation was performed according to protocols that were previously described with some modifications[11-13]. Dermal fibroblasts were suspended and lysed in an ice-cold buffer (20 mM Tris-HCl, pH 7.4, 150 mM NaCl, 5 mM EDTA) containing 2% Triton X-114 (Sigma) and protease inhibitors (Complete mini, Roche) for 15 min, followed by centrifugation at 15,000 x g at 4ºC and then precipitation of the supernatant with cold (-80ºC) acetone. The pellet was resuspended with buffer A (20 mM Tris-HCl, pH 7.4, 0.1% Triton X-100 and Complete Mini) either in the presence or absence of 1 unit/mL recombinant phosphatidylinositol-specific phospholipase C (PI-PLC, Invitrogen), followed by incubation for 120 min at 37ºC. Triton X-114 was added to the reaction to achieve a final Triton X-114 concentration of 2%, followed by additional incubation for 10 min at 37ºC and centrifugation (1,000 x g) for 5 min at 37ºC to separate upper aqueous (A) and lower detergent (D) phases. The detergent phase was resuspended in buffer A, incubated for 2 min at 37 °C, followed by centrifugation for 5 min at 37ºC to ensure the purity of the detergent phase. The proteins of the aqueous and the second detergent phases were analyzed by Western blot analysis.

Biotin labeling and isolation of cell surface proteins were done with the Cell Surface Protein Isolation Kit (Life Technologies), following the manufacturer's protocol. Extraction of nuclear proteins from C3H10T1/2 cells was done with ProteoExtract Subcellular Proteome Extraction Kit (Millipore).

Quantitative PCR

For the analysis of gene expression in cultured BM-MSCs, C3H10T1/2 cells or bone marrow stromal cells, total RNA was extracted using RLT buffer (Qiagen) and the RNeasy Mini Kit (Qiagen) and was treated with RNase-free DNase (Qiagen) according to the manufacturer's instructions. Purified RNA samples were reverse-transcribed by using ReverTra Ace (Toyobo, Tokyo, Japan) with oligo dT and random primers. Quantitative RT-PCR (qPCR) on the generated cDNAs was performed on an Mx3005P thermal cycler (Agilent Technologies). TaqMan probes and primers for human Meflin (*ISLR*) (Hs01921558_s1), mouse Meflin (*Islr*) (Mm01700423_m1), human *GAPDH* (Hs02758991_g1), mouse *Acan* (aggrecan, Mm00545794-m1), mouse *Col2a1* (Mm01309565_m1), mouse *Sox9* (Mm00448840_m1), mouse *Cxcl12* (Mm00445552_m1), mouse *Lepr* (Mm00440181_m1), and mouse *Gapdh* (Mm99999915_g1) were purchased from Life Technologies and used per the manufacturer's instructions. The data were analyzed using the comparative threshold cycle (CT) method and normalized against GAPDH controls.

For the analysis of gene expression in the long bones, tibiae were collected from wild-type and Meflin-deficient P70 mice. After the removal of the bone marrow by flushing, bone tissues were disrupted and homogenized using Multi-Beads Shocker (Yasui Kikai Corporation, Osaka, Japan) before RNA extraction. Total RNA extracted using TRIzol reagent (Invitrogen) and the RNeasy Mini Kit were treated with RNase-free DNase (Qiagen) according to the manufacturer's

instructions. Purified RNA samples were reverse-transcribed by using ReverTra Ace. qPCR analysis was performed using the MX3005P thermal cycler with SYBR Green PCR Master Mix according to the instructions of the manufacturer (Life Technologies). The PCR reactions were performed under the following cycling conditions: 95°C for 15 sec, annealing at 60°C for 30 sec, and extension at 72°C for 60 sec for 40 cycles. The data were analyzed by the comparative threshold cycle (CT) method and normalized against *Gapdh* controls. Sequences of the primers areas were as follows: *Gapdh* forward, 5'-AGTATGACTCCACTCACGGCAA-3'; *Gapdh* reverse 5'-TCTCGCTCCTGGAAGATGGT-3'; *Runx2* forward, 5'-TTGACCTTTGTCCCAATGC-3'; *Runx2* reverse, 5'-AGGTTGGAGGCACACATAGG-3'; Osteocalcin forward, 5'-GCAATAAGGTAGTGAACAGACTCC-3'; Osteocalcin reverse, 5'-AGCAGGGTTAAGCTCACACTG-3'; Osteonectin forward, 5'-ATTTGAGGACGGTGCAGAGG-3'; Osteonectin reverse, 5'-TCTCGTCCAGCTCACACACCT-3'; Collagen 1a1 forward, 5'-GAAGGCAACAGTCGATTCACC-3'; Collagen 1a1 reverse, 5'-GACTGTCTTGCCCCAAGTTCC-3'.

***In situ* hybridization (ISH)**

Paraffin-embedded blocks and tissue sections of E18.5 and P56 C57BL/6 mice were obtained from Genostaff (Tokyo, Japan). The mouse tissues were dissected, decalcified, treated with Tissue Fixative (Genostaff) and embedded in paraffin with their proprietary procedures, followed by sectioning at 4 - 8 µm. For ISH, tissue sections were dewaxed with xylene and rehydrated through an ethanol series and a PBS wash. The sections were fixed by 4% paraformaldehyde treatment for 15 min, washed with PBS, treated with Proteinase K (8 - 10 µg/mL) in PBS for 30 min at 37ºC, washed with PBS, refixed with 4% paraformaldehyde, washed with PBS, and then placed in 0.2N HCl for 10 min. After washing with PBS, the sections were acetylated by incubation in 0.1M triethanolamine-HCl (pH 8.0, 0.25% acetic anhydride) for 10 min. After washing with PBS, the sections were dehydrated through a series of ethanol rinses. Hybridization was performed with probes, the sequences of which are described in Supplemental Information, at concentrations of 300 ng/mL in the Probe Diluent-1 (Genostaff) for 16 h at 60ºC. After hybridization, the sections were washed in 5 x HybriWash (Genostaff) for 20 min at 60ºC and 50% formamide and 2 x HybriWash for 20 min at 60ºC, followed by RNase treatment (50 µg/mL RNaseA, 10 mM Tris-HCl, pH 8.0, 1 M NaCl and 1 mM EDTA) for 30 min at 37ºC. Next, the sections were washed twice with 2 x HybriWash for 20 min at 60ºC, twice in 0.2 x HybriWash for 20 min at 60ºC, and once with TBST (0.1% Tween20 in Tris-buffered saline). After treatment with 1 x G-Block (Genostaff) for 15 min at room temperature (RT), the sections were incubated with anti-digoxigenin–alkaline phosphatase (DIG AP) conjugate (Roche) diluted 1:2000 with 0.02 x G-Block in TBST for 1 h at RT. The sections were washed twice with TBST and incubated in 100 mM NaCl, 50 mM $MgCl_2$, 0.1% Tween20, 100 mM Tris-HCl, pH 9.5. Coloring reactions were performed with NBT/BCIP

solution (Sigma) overnight and washed with PBS. The sections were counterstained with Kernechtrot stain solution (Mutoh, Tokyo, Japan) and mounted with CC/Mount (DBS). Sequences of probes used for ISH were as follows:

Probe 1 (AS-1, Sense-1) (Sequence Position: 725-1439)

GCCTCAGCGGTGTCTATTCCAGAACAGGACAACATTGCCTGTACTACACCCCACGTCCTGA
AGGGTATCCCACTAGGCCGCCTGCCACCCCTGCCCTGCTCAGCTCCCTCAGTGCAACTAA
GCTACCAGCCCAGCCAAGATGGAGCAGAGCTACGGCCTGGCTTCGTGCTGGCACTCCACT
GTGACGTGGATGGACAGCCAGTCCCCCAGCTCCATTGGCACATTCACACCCCGGGCGGCA
CGGTGGAGATCGCCAGTCCTAATGTAGGCACTGATGGACGTGCCCTGCCTGGTGCCCTTG
CAACCAGTGGGCAGCCACGCTTCCAGGCCTTTGCCAATGGCAGCCTGCTTATCCCTGACTT
TGGCAAGCTGGAGGAGGGCACCTATAGCTGCCTGGCCACCAATGAGCTAGGCAGTGCCG
AAAGCTCTGTAAATGTGGCATTGGCCACCCCAGGTGAGGGGGGAGAGGATGCTGTGGGG
CACAAGTTCCATGGCAAAGCAGTGGAGGGCAAGGGCTGCTATACGGTTGACAACGAGGTA
CAGCCATCCGGACCGGAAGACAACGTGGTTATCATTTACCTCAGCCGTGCTGGGCCCCCA
GAAGCTGCAATAGCAGCAGACGGGAGGCCTGCGCAGCAGTTCTCTGGCATACTTCTGCTA
GGCCAAAGCCTGCTTGTTCTCTCCTTTTTCTACTTCTAACTACACCCTGCCCTGG

Probe 2 (AS-2, Sense-2) (Sequence Position: 1333-2026)

AATAGCAGCAGACGGGAGGCCTGCGCAGCAGTTCTCTGGCATACTTCTGCTAGGCCAAAG
CCTGCTTGTTCTCTCCTTTTTCTACTTCTAACTACACCCTGCCCTGGTTGGCTAGAGCAGCT
CCAGGGCCTTCCTAACTCCCCCTGATCACGTTCCTGCCAATGTTCCGTCTTAACACCACGA
GGTCTGGAATTGGTGAGGCCTGAGGTTAGCCTGGGGACTTCACATTTTCCTATCACCTTTT
CTAAGCTCATCCAGGCCGCTCATTACTCCAATTTGCAGATCTGCTCAGAACTAGCAGCTAG
GATAGAACTATATCCCAAAACTCATCATCTCTAGTGCTAGTTGCTGCTAACAGCATTGCCTG
TGCTCCTAGCAGGGGCAGCCTGCTAACAGGGCAACAACAATGTCTTAACCTGACCTACTTT
GAGAGTTCTAGCCATGGAGGTAAAGAGCTTGTGGAGGCCATCCAGGTGGGCACTGGGGCT
GGGCTAGAAGGGGGTCTGGAGTGGCCAGTACAGGATCTGGAAAGGAGGTACCACGGGCC
ATGGCTGGGCAGGCTAAAAGCTTTCCTGTTTTTAGATGCTCTCTGATGAGATATGGAAATCA
TCCCTCACTGGGCCTTTCTCTCCTCTCACCCCAAGCCCCACAGCATGTGCCTGCCTCTTGA
GACACTTACTCCCCCTCCTCCTCT

Probe 3 (AS-3, Sense-3) (Sequence Position: 725-2026)

GCCTCAGCGGTGTCTATTCCAGAACAGGACAACATTGCCTGTACTACACCCCACGTCCTGA
AGGGTATCCCACTAGGCCGCCTGCCACCCCTGCCCTGCTCAGCTCCCTCAGTGCAACTAA
GCTACCAGCCCAGCCAAGATGGAGCAGAGCTACGGCCTGGCTTCGTGCTGGCACTCCACT
GTGACGTGGATGGACAGCCAGTCCCCCAGCTCCATTGGCACATTCACACCCCGGGCGGCA
CGGTGGAGATCGCCAGTCCTAATGTAGGCACTGATGGACGTGCCCTGCCTGGTGCCCTTG
CAACCAGTGGGCAGCCACGCTTCCAGGCCTTTGCCAATGGCAGCCTGCTTATCCCTGACTT

TGGCAAGCTGGAGGAGGGCACCTATAGCTGCCTGGCCACCAATGAGCTAGGCAGTGCCG
AAAGCTCTGTAAATGTGGCATTGGCCACCCCAGGTGAGGGGGAGAGGATGCTGTGGGG
CACAAGTTCCATGGCAAAGCAGTGGAGGGCAAGGGCTGCTATACGGTTGACAACGAGGTA
CAGCCATCCGGACCGGAAGACAACGTGGTTATCATTTACCTCAGCCGTGCTGGGCCCCCA
GAAGCTGCAATAGCAGCAGACGGGAGGCCTGCGCAGCAGTTCTCTGGCATACTTCTGCTA
GGCCAAAGCCTGCTTGTTCTCTCCTTTTTCTACTTCTAACTACACCCTGCCCTGGTTGGCTA
GAGCAGCTCCAGGGCCTTCCTAACTCCCCCTGATCACGTTCCTGCCAATGTTCCGTCTTAA
CACCACGAGGTCTGGAATTGGTGAGGCCTGAGGTTAGCCTGGGGACTTCACATTTTCCTAT
CACCTTTTCTAAGCTCATCCAGGCCGCTCATTACTCCAATTTGCAGATCTGCTCAGAACTAG
CAGCTAGGATAGAACTATATCCCAAAACTCATCATCTCTAGTGCTAGTTGCTGCTAACAGCA
TTGCCTGTGCTCCTAGCAGGGGCAGCCTGCTAACAGGGCAACAACAATGTCTTAACCTGAC
CTACTTTGAGAGTTCTAGCCATGGAGGTAAAGAGCTTGTGGAGGCCATCCAGGTGGGCAC
TGGGGCTGGGCTAGAAGGGGGTCTGGAGTGGCCAGTACAGGATCTGGAAAGGAGGTACC
ACGGGCCATGGCTGGGCAGGCTAAAAGCTTTCCTGTTTTTAGATGCTCTCTGATGAGATAT
GGAAATCATCCCTCACTGGGCCTTTCTCTCCTCTCACCCCAAGCCCCACAGCATGTGCCTG
CCTCTTGAGACACTTACTCCCCCTCCTCCTCT

Immunohistochemistry

For the analysis of the colocalization of Meflin and the leptin receptor in BM, paraffin-embedded femur and tibia (knee joint) tissues prepared from P56 C57BL/6 mice (Genostaff) were serially sectioned and used for Meflin ISH using the probe AS-2 and immunohistochemistry (IHC) for the leptin receptor. For leptin receptor staining, the sections were deparaffinized with xylene and rehydrated with PBS, followed by antigen retrieval by microwaving in a citric acid buffer (pH 6). After washing with PBS, the sections were treated with 0.3% H_2O_2 in PBS for 30 min and washed with TBS, followed by blocking with G-Block (Genostaff) and Avidin/Biotin Blocking Kit (Vector laboratories). The sections were then incubated with anti-leptin receptor goat polyclonal antibody (R&D systems, AF497, 0.4 µg/mL) overnight at 4ºC, washed with TBS and incubated with biotin-conjugated anti-goat Ig (Dako) for 30 min, followed by the addition of peroxidase-conjugated streptavidin (Nichirei, Japan) for 5 min and signal detection with diaminobenzidine solution.

Luciferase reporter assay

pGL4.10-luc2 luciferase vector (Promega) harboring the human *Sox9* promoter region (-1068 - +1, PGL4.10-Sox9) was generously provided by Bisei Ohkawara and Kinji Ohno (Nagoya University). Human BM-MSCs seeded in 6-cm culture dishes were transfected with the pGL4.10-Sox9 (3 µg) as well as phRL-TK encoding *Renilla* luciferase (Promega, 1 µg) using the X-tremeGene HP DNA Transfection Reagent (Roche) according to the manufacturer's protocols. After 48 h of transfection,

luciferase activity was measured using the Dual Luciferase Reporter Assay System (Promega) and LB 9508 Lumat3 tube luminometer (Berthold Technologies). Firefly luciferase activity was normalized to *Renilla* luciferase activity and relative luciferase units (RLU) are indicated.

Measurement of alkaline phosphatase (ALP) activity and Alizarin red staining

We performed ALP activity assays with the ALP Assay Kit (TaKaRa Bio, Shiga, Japan) following the manufacturer's protocol. For the detection of mineralization, we induced osteogenic differentiation of C3H10T1/2 cells for 2 weeks, followed by fixation of the cells with neutral-buffered formalin and staining with the alizarin red solution for 30 min. The cells were then incubated with calcified nodule extraction solution (Cosmobio) with shaking for 10 min. The absorbance of the extracted solution was measured at 405 nm.

Culture on elastic silicone substrates

Elastically supported surface dishes (μ-Dish, 35-mm, high ESS) with stiffness values of 1.5, 15 or 28 kPa were purchased from ibidi (Martinsried, Germany). Polydimethylsiloxane (PDMS) substrate with a stiffness of 2 MPa was prepared using a Sylgard 184 silicone elastomer kit (Dow Corning). The silicone elastomer component was mixed with the curing agent (elastomer to curing agent ratio: 10:1), degassed, and coated on glass bottom dishes (ibidi). Subsequently, crosslinking of the elastomer was carried out at 70ºC for 4 h. The surface of substrates was silanized by 3-aminopropyltriethoxysilane via gas-phase reaction, modified with glutaraldehyde and functionalized overnight with 0.2% gelatin and 2 μg/mL fibronectin. The surface was then extensively washed with PBS and cell culture medium.

Gene expression of mouse Meflin and GAPDH was determined by qPCR using a LightCycler Nano (Roche Diagnostics) on cDNA samples by use of FastStart Essential DNA Green Master (Roche Diagnostics). DNA levels during the linear phase of amplification were normalized against GAPDH controls. Calculation were made in triplicate and expressed as means ± S.D. Primer sequences: Meflin primer 1 forward, 5'-ACTTGCGAGCAATCCAGTCCTTA -3'; Meflin primer 1 reverse, 5'-ACAGTCTGCAATCTGGAAGCCATAC -3'; Meflin primer 2 forward, 5'-GGCACAAGTTCCATGGCAAAG -3'; Meflin primer 2 reverse, 5'-CACGGCTGAGGTAAATGATAACCAC-3'.

Production and purification of recombinant Meflin

Recombinant mouse Meflin was produced by the silkworm expression system (ProCube, Sysmex, Kobe Japan) as described previously[14-17]. Mouse Meflin tagged with a secretion signal sequence (SP) and a Flag tag at the N-terminus (SP-Flag-Meflin) was subcloned into baculoviral expression vector pM01 (Sysmex) harboring the polyhedrin promoter, which was then cotransfected into BmN (*B. mori*) cells with baculovirus genomic DNA to produce recombinant virus. After propagation of the recombinant baculovirus in BmN cells, the virus was infected into silkworm larvae of the fifth instar early stage. Five days after inoculation, the hemolymph of the larvae containing recombinant Meflin was collected and centrifuged at 100,000 x g for 1 h. The supernatants (20 mL) were subjected to affinity purification using anti-Flag agarose followed by elution with the Flag peptide.

Mouse genotyping

Genomic DNAs extracted from mouse tails were used for PCR genotyping. Sequences of the primers are as follow: PCR1 forward, 5'-GCTGCATTTGAGCTGAGCCTCTGG-3'; PCR1 reverse, 5'-AACCCCTTCCTCCTACATAGTTGG-3'; PCR2 forward, 5'-TGAGGTTAGCCTGGGGACTTCAC-3'; PCR2 reverse, 5'-GGCTAGAACTCTCAAAGTAGGTCAGG-3'.

Analysis of mouse skeleton

Skeletal preparations were made following the method described previously[18]. Fetuses were skinned, eviscerated, and dehydrated in 90% ethanol for 4 days and kept in acetone for another 2 days, followed by incubation in staining solution (1 vol. of 0.3% Alcian blue in 70% ethanol, 1 vol. of 0.1% alizarin red S in 95% ethanol, 1 volume of acetic acid, and 17 volumes of 70% ethanol) at 37°C for 4 days. The samples were rinsed in water and kept in 1% potassium hydroxide overnight at room temperature, followed by incubation in 1% potassium hydroxide/20% glycerol overnight at room temperature. The samples were then transferred into 50%, 80%, and 100% glycerol, and then photographed with a stereomicroscope (Olympus SZX7).

Bone histo-morphometric analysis

The tibiae of 10-week-old wild-type (n = 4) and Meflin-deficient (n = 5) male mice were subjected to histo-morphometric analysis at Kureha Special Laboratory (Tokyo, Japan). Briefly, bones were fixed with 70% ethanol, embedded (without decalcification) in carboxymethylcellulose, and sectioned longitudinally into 5-μm slices using a cryostat (Leica CM3050S; Leica Microsystems, Wetzlar, Germany), followed by staining with toluidine blue. The static parameters of the trabecular bones were determined in a defined area of the secondary spongiosa between 0.25 and 1.15 μm

below the growth plate at ×400 magnification, using an Olympus BX51 light microscope (Tokyo, Japan) and the OsteoMeasure morphometry system (Osteometrics, Atlanta, GA, USA). The terminology and units used are those recommended by the Histo-morphometry Nomenclature Committee of the American Society for Bone and Mineral Research. The width of the growth plate and the proliferative zone was determined on images at ×200 magnification, which were obtained at nine different points within a central undisturbed region of 1.8 mm width equidistant from the lateral edges of the bone.

Isolation of BM-MSCs from compact bones and colony-forming unit fibroblast (CFU-F) assays

For the isolation of BM-MSCs, we followed the protocol for the isolation of mesenchymal progenitors from mouse compact bone provided by StemCell Technologies. Briefly, the femurs and tibiae collected from wild-type and Meflin-deficient P56 mice were cleared of epiphyses and soft tissue and crushed using a meno mortar and pestle with PBS supplemented with 2% FBS, 1 mM EDTA, and penicillin and streptomycin (Life Technologies). The BM suspension was filtered through a 70 µm cell strainer (BD Biosciences) to remove bone debris, followed by treatment with 0.25% collagenase type I in PBS containing 20% FBS. The cell suspension was enriched immuno-magnetically for $CD45^{-}TER119^{-}$ cells (negative selection) using the EasySep Mouse Mesenchymal Progenitor Enrichment Kit For Compact Bone and EasySep Magnet (StemCell Technologies) according to the manufacturer's instructions. The collected enriched cells were plated on 10-cm dishes at a density of 2,000 cells/dish in MesenCult MSC Basal Medium supplemented with MesenCult Mesenchymal Stem Cell Stimulatory Supplements (StemCell Technologies). The cells were incubated for two weeks without changing medium, and stained with Giemsa solution, followed by counting fibroblastic colonies in each dish. Colonies containing more than 50 cells were counted after Giemsa coloration.

Flow cytometry and sorting

Isolation of mouse MSCs was performed as described previously[19]. BM suspensions isolated from the femurs and tibiae of C57BL/6 mice (8 - 10 weeks) were hemolyzed with Lysing Buffer (BD Biosciences) to remove the red blood cells, followed by enrichment for $CD45^{-}TER119^{-}$ cells using the EasySep Mouse Mesenchymal Progenitor Enrichment Kit. The cells were resuspended in staining medium (PBS with 2% FBS and 1mM EDTA) including anti-CD16/32 antibody (BD Bioscience) to block Fc receptors for 5 min at 4ºC, followed by staining with fluorochrome-conjugated antigen-specific antibodies or isotype control antibodies on ice for 30 min. The antibodies used in this study to identify MSCs were as follows: anti-Sca-1-PE (BioLegend, clone D7, 1:40), anti-CD140a (PDGFRα)-APC (eBioscience, clone APA5, 1:40), anti-CD140b

(PDGFRβ)-APC (eBioscience clone APB5, 1:40), anti-CD45-PerCP-Cy5.5 (Biolegend, clone 30-F11, 1:200) and anti-CD31-FITC (Biolegend, clone 390, 1:100). The cells were analyzed on a flow cytometer FACSCanto (BD Bioscience) or sorted using a FACSAria II (BD Biosciences). Debris and dead cells were excluded by forward and side scatter.

For the isolation of PDGFRα$^+$ cells from skeletal muscle, we followed the protocol previously described by Uezumi et al[20]. Hind limb muscles of C57BL/6 wild-type 8- to 10-week-old mice were excised, followed by digestion of the trimmed muscles with 0.2% type II collagenase (Worthington) for 60 min at 37ºC. The digested muscles were filtered through a 70 μm cell strainer. Erythrocytes were eliminated by adding Lysing Buffer (BD Biosciences), followed by enrichment for CD45$^-$TER119$^-$ cells using the EasySep Mouse Mesenchymal Progenitor Enrichment Kit. The cells were suspended in buffer consisting of PBS with 2% FBS, followed by the analysis on a FACSCanto flow cytometer and sorting on a FACSAria II.

Analysis of peripheral blood

Mice were anesthetized with isoflurane and peripheral blood samples collected from the retro-orbital sinus with heparinized capillary tubes. Blood counts were performed using a KX-21 hematology analyzer (Sysmex, Kobe, Japan). For FACS analysis, 30-50 μl of PB was treated with red cell lysis buffer, and then stained with anti-CD3e(eBioscience, clone 145-2C11), anti-CD11b (eBioscience, clone M1/70), anti-CD19 (eBioscience, clone eBio1D3) , anti-CD45R/B220 (eBioscience, clone RA3-6B2) and anti-Ly6G (eBioscience, clone RB6-8C5) . FACS analysis was performed on FACS Aria flow cytometer (BD Biosciences), followed by analysis with FlowJo software (Tree Star).

Data analysis

Data are presented as the means ± S.D. Statistical significance was evaluated with Student's *t* test.

Supplemental References

1. Chambers, S.M. *et al.* Hematopoietic fingerprints: an expression database of stem cells and their progeny. *Cell Stem Cell* **1**, 578-591 (2007).

2. Ding, L., Saunders, T.L., Enikolopov, G. & Morrison, S.J. Endothelial and perivascular cells maintain haematopoietic stem cells. *Nature* **481**, 457-462 (2012).

3. Bianco, P. & Robey, P.G. Skeletal stem cells. *Development* **142**, 1023-1027 (2015).

4. Kfoury, Y. & Scadden, D.T. Mesenchymal cell contributions to the stem cell niche. *Cell Stem Cell* **16**, 239-253 (2015).

5. Morrison, S.J. & Scadden, D.T. The bone marrow niche for haematopoietic stem cells. *Nature* **505**, 327-334 (2014).

6. Mendelson, A. & Frenette, P.S. Hematopoietic stem cell niche maintenance during homeostasis and regeneration. *Nature Medicine* **20**, 833-846 (2014).

7. Worthley, D.L. *et al.* Gremlin 1 identifies a skeletal stem cell with bone, cartilage, and reticular stromal potential. *Cell* **160**, 269-284 (2015).

8. Caplan, A.I. & Correa, D. The MSC: an injury drugstore. *Cell Stem Cell* **9**, 11-15 (2011).

9. Haga, K. *et al.* Permanent, lowered HLA Class I expression using lentivirus vectors with shRNA constructs: averting cytotoxicity by alloreactive T lymphocytes. *Transplantation Proceedings* **38,** 3184-3188 (2006).

10. Enomoto, A. *et al.* Roles of disrupted-in-schizophrenia 1-interacting protein girdin in postnatal development of the dentate gyrus. *Neuron* **63**, 774-787 (2009).

11. Kramer, E.M., Koch, T., Niehaus, A. & Trotter, J. Oligodendrocytes direct glycosyl phosphatidylinositol-anchored proteins to the myelin sheath in glycosphingolipid-rich complexes. *The Journal of Biological Chemistry* **272**, 8937-8945 (1997).

12. Fernández-Messina, L., Ashiru, O., Agüera-González, S., Reyburn, H.T. & Valés-Gómez, M. The human NKG2D ligand ULBP2 can be expressed at the cell surface with or without a GPI anchor and both forms can activate NK cells. *Journal of Cell Science* **124**, 321-327 (2011).

13. Chen, L.M. *et al.* Prostasin is a glycosylphosphatidylinositol-anchored active serine protease. *The Journal of Biological Chemistry* **276**, 21434-21442 (2001).

14. Nagaya, H. Recombinant protein production by a Kaiko-baculovirus system. *Methods in Molecular Biology (Clifton, N.J.)* **577**, 109-120 (2009).

15. Shin, J., Kim, M.A., Kobayashi, M. & Sohn, Y.C. Production and characterization of recombinant Manchurian trout thyrotropin. *Fish Physiology and Biochemistry* **39**, 1353-1363 (2013).

16. Ujita, M. *et al.* Expression of active and inactive recombinant soluble trehalase using baculovirus-silkworm expression system and their glycan structures. *Journal of Bioscience and Bioengineering* **111**, 22-25 (2011).

17. Kobayashi, M. *et al.* Production of recombinant Japanese eel gonadotropins by baculovirus in silkworm larvae. *General and Comparative Endocrinology* **167**, 379-386 (2010).

18. Wallin, J. *et al.* The role of Pax-1 in axial skeleton development. *Development* **120**, 1109-1121 (1994).

19. Houlihan, D.D. *et al.* Isolation of mouse mesenchymal stem cells on the basis of expression of Sca-1 and PDGFR-α. *Nature Protocol* **7,** 2103-2111 (2012).

20. Uezumi, A. et al. Mesenchymal progenitors distinct from satellite cells contribute to ectopic fat cell formation in skeletal muscle. *Nature Cell Biology* **12,** 143-152 (2010).
